# A First-In-Human Study of the SUMOylation Inhibitor Subasumstat in Patients with Advanced/Metastatic Solid Tumors or Relapsed/Refractory Hematologic Malignancies

**DOI:** 10.1158/2767-9764.CRC-25-0243

**Published:** 2025-11-19

**Authors:** Dejan Juric, Daniel Anderson, Afshin Dowlati, Jordi Rodon, Iwona Lugowska, Sławomir Mańdziuk, Yuqin Song, Feng Jung Sherida H. Woei-A-Jin, Marc André, Joanna Góra Tybor, José-Ángel Hernández-Rivas, Razelle Kurzrock, Armando López-Guillermo, David Schröder, Rafal Stec, Allison Berger, Bo Chao, Aleksander Chudnovsky, John P. Gibbs, Tao Long, Dina Stroopinsky, Qi Dong, Anthony J. Olszanski

**Affiliations:** 1 Termeer Center for Targeted Therapies, Massachusetts General Hospital, Boston, Massachusetts.; 2 HealthPartners Oncology, HealthPartners Institute, Minneapolis, Minnesota.; 3 Department of Medicine, University Hospitals Seidman Cancer Center, Case Western Reserve University, Cleveland, Ohio.; 4 Investigational Cancer Therapeutics, The University of Texas MD Anderson Cancer Center, Houston, Texas.; 5 Centre for Excellence in Precision Oncology, Maria Skłodowska-Curie National Research Institute of Oncology, Warsaw, Poland.; 6 Medical University of Lublin, Lublin, Poland.; 7 Department of Lymphoma, Peking University Cancer Hospital & Institute (Beijing Cancer Hospital), Beijing, China.; 8 Department of General Medical Oncology, Leuven Cancer Institute, University Hospitals Leuven, KU Leuven, Leuven, Belgium.; 9 Hematology Department, CHU UCL Namur, Yvoir, Belgium.; 10 Hematology, Institute of Hematology and Blood Transfusion, Warsaw, Poland.; 11 Hematology Department, Infanta Leonor University Hospital, Complutense University, Madrid, Spain.; 12 WIN Consortium for Precision Medicine, Chevilly-Larue, France.; 13 Medical College of Wisconsin, Milwaukee, Wisconsin.; 14 Hematology Department, Hospital Clinic Barcelona, Barcelona, Spain.; 15 Medical Oncology Department, Grand Hôpital de Charleroi, Charleroi, Belgium.; 16 Department of Oncology, Warsaw Medical University, Warsaw, Poland.; 17 Biokinetica, Józefów, Poland.; 18 Oncology Therapeutic Area Unit, Takeda Development Center Americas, Inc. (TDCA), Cambridge, Massachusetts.; 19 Takeda Development Center Americas, Inc. (TDCA), Cambridge, Massachusetts.; 20 Oncology Clinical Development, Takeda Development Center Americas, Inc. (TDCA), Cambridge, Massachusetts.; 21 Quantitative Clinical Pharmacology, Takeda Development Center Americas, Inc. (TDCA), Cambridge, Massachusetts.; 22 Clinical Science, Takeda Development Center Americas, Inc. (TDCA), Cambridge, Massachusetts.; 23 Medical Oncology, Fox Chase Cancer Center, Philadelphia, Pennsylvania.

## Abstract

**Purpose:**

Subasumstat (TAK-981) is a first-in-class inhibitor of SUMOylation that can engage innate and adaptive immune responses in tumors by enhancing type I IFN (IFNI) production. We conducted a phase I/II dose-escalation/-expansion study (NCT03648372) to investigate the safety, pharmacokinetics, pharmacodynamics, and preliminary efficacy of subasumstat as a single agent in patients with advanced/metastatic solid tumors and relapsed/refractory hematologic malignancies.

**Patients and Methods:**

Eligible patients received subasumstat intravenously at escalating doses twice weekly (days 1, 4, 8, and 11) or once weekly (days 1 and 8) in 21-day cycles until disease progression or unacceptable toxicity.

**Results:**

A total of 109 patients were enrolled (solid tumors: *n* = 100; lymphomas, *n* = 9). In phase I, four patients reported dose-limiting toxicities of grade 3 alanine transaminase/aspartate transaminase elevation, pneumonitis, stomatitis, and cognitive disorder; 120 mg twice weekly was determined as the MTD. The most common adverse events were fatigue (47%), nausea (41%), diarrhea (36%), and pyrexia (36%). Pharmacodynamic analyses demonstrated target engagement and SUMOylation pathway inhibition, induction of an IFNI-regulated gene signature and cytokine production, and activation of innate and adaptive immune cells. Based on safety, pharmacokinetic, and pharmacodynamic findings, 90 mg twice weekly was proposed as the recommended phase II dosage. Overall, three and 26 patients achieved a partial response and stable disease, respectively.

**Conclusions:**

Subasumstat had a manageable safety profile, with evidence of innate and adaptive immune response engagement in patients with advanced/metastatic solid tumors and relapsed/refractory hematologic malignancies. Further studies are needed to determine the role of subasumstat in cancer treatment.

**Significance:**

Identification of novel and effective therapies for patients who become refractory to standard anticancer treatments remains paramount. In this phase I/II study, subasumstat, a first-in-class SUMOylation inhibitor, had preliminary clinical activity and demonstrated target engagement, upregulation of IFN and plasma cytokines, and activation of innate and adaptive immune cells.

## Introduction

SUMOylation is a reversible posttranslational modification in which small ubiquitin-like modifier (SUMO) proteins are activated and covalently attached to substrate proteins (1, 2). SUMO1 conjugates to proteins as a monomer, whereas SUMO2 and SUMO3 attach as oligomeric chains (3). Dysregulation of SUMOylation patterns has been observed in various solid and hematologic tumors (4–8), and proteomic studies have identified several thousand SUMOylated proteins, with roles in critical cellular processes, including gene expression, DNA damage response, RNA processing, and cell-cycle progression (9). Many tumors show upregulation of SUMOylation pathway components at the mRNA or protein level, such as in lung (10, 11), colorectal (12), or pancreatic (13) cancers. In addition, SUMOylation plays a key role in normal immune cells by repressing gene expression of IFNβ, a type I IFN (IFNI; ref. 2).

Subasumstat is an intravenous, first-in-class, small-molecule inhibitor of the SUMO-activating enzyme (SAE), which is required for all SUMOylation (Supplementary Fig. S1; ref. 14). The mechanism of enzyme inhibition involves the simultaneous binding of a SUMO protein and the subasumstat inhibitor in the two pockets of the active site and catalytic formation of a SUMO–subasumstat adduct, which can be detected by an anti-adduct antibody.

In preclinical experiments, SUMOylation inhibition (assessed via the presence of SUMO–subasumstat adduct and by a decrease in SUMO2/3 protein conjugates using IHC) promoted endogenous IFNI production in immune cells, leading to maturation and activation of multiple immune cell types, including antigen-presenting dendritic cells, NK cells, macrophages, and T cells (2, 15, 16). *In vivo* studies in xenograft models of lymphoma and pancreatic cancer demonstrated that subasumstat inhibits SUMOylation in tumors and shows antitumor activity in immunocompromised mice (14), while also demonstrating a notable pharmacodynamic effect in the immune system of immunocompetent mice, with IFNI pathway activation and associated immune-dependent antitumor activity (15, 17). In *ex vivo* macrophages and NK cells, treatment with subasumstat led to IFNI-dependent innate immune activation and enhanced killing of tumor cell targets (16), whereas in immunocompetent mice, subasumstat was shown to enhance antigen cross-presentation and promote T- and NK-cell activation through increases in CD69 and CD80/CD86 surface proteins, respectively (15, 18). No systemic toxicities were observed in the preclinical mouse models following subasumstat administration (15–17), with transient lymphopenia seen in A20 lymphoma–bearing BALB/c mice (consistent with a response to IFNI; ref. 15) and transient B-cell depletion in a mouse model of pancreatic ductal adenocarcinoma (17).

Based on these promising preclinical data, we conducted a first-in-human study (NCT03648372) of single-agent subasumstat in patients with advanced or metastatic solid tumors or relapsed/refractory hematologic malignancies.

## Patients and Methods

### Patients

Eligible patients were aged ≥18 years, with an Eastern Cooperative Oncology Group performance status of 0 to 1. In phase I dose escalation, eligible patients either had a histologically or cytologically confirmed advanced or metastatic solid tumor for which no standard treatment option with proven clinical benefit was available or a relapsed/refractory lymphoma not amenable to therapies with proven clinical benefit or that failed at least two prior systemic therapies. Patients eligible for phase II dose expansion had a histologically or cytologically documented advanced (metastatic and/or unresectable) cancer that was incurable and for which prior standard first-line treatment had failed, including non-squamous non–small cell lung cancer (NSCLC) that had progressed following no more than two lines of therapy, including a prior checkpoint inhibitor (CPI)–containing therapy; CPI-naïve cervical cancer with no more than one prior line of systemic therapy; CPI-naïve microsatellite-stable (MSS) colorectal cancer that had progressed on no more than three prior lines of therapy; relapsed/refractory diffuse large B-cell lymphoma (DLBCL) after prior chimeric antigen receptor T cell (CAR-T) therapy; relapsed/refractory DLBCL following at least two, but no more than three, prior lines of systemic therapy; and relapsed/refractory follicular lymphoma (FL) after at least two, but no more than three, prior lines of systemic therapy (with at least one including CD20-targeted therapy). The phase II dose-expansion solid tumor and lymphoma cohorts were designed to match those of two other phase I/II studies of subasumstat in combination with pembrolizumab (NCT04381650; ref. 18) and rituximab (NCT04074330; ref. 19), respectively. Full eligibility criteria for each phase of the study are described in the Supplementary Methods.

### Study design

**Figure 1.**
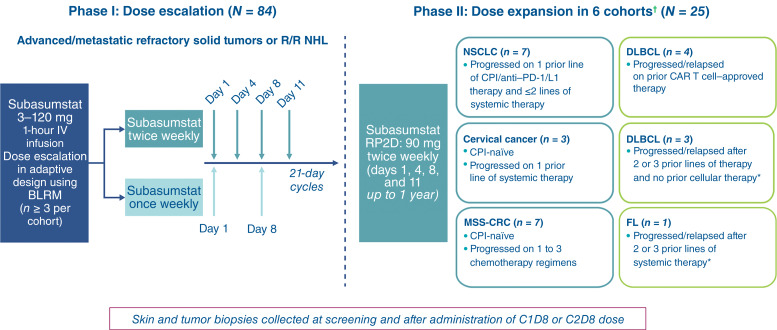
Study design. *, At least one line of prior therapy must have included a CD20-targeted therapy; †, each cohort will be expanded to 26 or 38 patients if interim futility analyses are passed. BLRM, Bayesian logistic regression modeling; CRC, colorectal cancer; IV, intravenous; NHL, non–Hodgkin lymphoma; R/R, relapsed/refractory.

This study comprised two parts: a phase I dose escalation and a phase II dose expansion (Fig. 1). In the dose-escalation phase, patients were enrolled to receive subasumstat intravenously as a 1-hour (±10 minutes) infusion on days 1, 4, 8, and 11 of a 21-day cycle (biweekly schedule). Two less-intensive schedules of days 1 and 8 (weekly ) and days 1, 8, and 15 (single cohort) were evaluated per protocol following supportive safety, pharmacokinetics (PK), and pharmacodynamic data on the biweekly schedule. Dose escalation was based on cohort with an adaptive design using Bayesian logistic regression modeling with overdose control. The starting dose of subasumstat was based on Good Laboratory Practice animal toxicity studies and supported by the estimation of a minimum anticipated biologically effective level. A detailed description of dose-limiting toxicities (DLT) is presented in the Supplementary Methods.

The phase II dose-expansion portion enrolled patients to receive the recommended phase II dose (RP2D) determined in phase I, with each cohort assessed separately using an adaptive two-stage design for a single proportion (see Supplementary Methods). For stage 1, each cohort was analyzed when a prespecified number of patients were enrolled and had the potential to have at least one posttreatment scan [i.e., after the first disease assessment, 2 months from cycle 1 day 1 (C1D1)]. If the prespecified minimal response rate was not achieved in the first stage for a given cohort, that cohort would be closed to enrollment. If the required response rate during stage 1 or a good clinical benefit was observed for a particular cohort as mentioned above, then additional patients would be enrolled in the second stage of the corresponding cohort until a predetermined number of additional patients for that cohort had been reached. However, if a clinical benefit was observed for patients in the cohort [e.g., most patients had recorded stable disease (SD) at week 8 and, per investigator assessment, were benefiting from treatment], then enrollment into stage 2 would be allowed for this cohort with agreement from participating investigators. The final analysis of the primary endpoints for each cohort would take place when all ongoing patients had the opportunity to complete the 6-month disease assessment. Across both phases, patients received subasumstat for up to 1 year or until confirmed disease progression, unacceptable toxicity, if any withdrawal criterion from the study was met, or if discontinuation of subasumstat occurred.

The study was conducted in accordance with the International Council for Harmonization Good Clinical Practice standards, the Declaration of Helsinki, and all applicable regulatory requirements. Relevant institutional review boards or ethics committees approved all aspects of the study, and all authors had access to primary clinical trial data. All patients provided written informed consent. The trial is registered at ClinicalTrials.gov (NCT03648372).

### Objectives

The phase I primary objectives were to determine the safety and tolerability and to establish an RP2D of subasumstat as a single agent in patients with advanced or metastatic solid tumors and lymphomas. Phase I secondary objectives included assessment of target engagement (SUMO–subasumstat adduct formation) and SUMOylation pathway inhibition (decrease in SUMO2/3 conjugated proteins) in skin and peripheral blood cells and characterization of the subasumstat PK profile. The phase II primary objectives were to evaluate the preliminary efficacy of subasumstat in patients with select solid tumors or relapsed/refractory CD20^+^ non–Hodgkin lymphoma indications (except for marginal zone lymphoma). Safety and tolerability of subasumstat were a secondary objective in phase II.

Exploratory objectives for both phase I and phase II included the assessment of SUMO–subasumstat adduct formation and SUMOylation pathway inhibition in blood, skin, and tumors; induction of the IFNI gene expression signature in blood; and changes in T and NK cells in blood and paired tumor biopsies.

### Study assessments

The occurrence of DLTs was assessed during cycle 1 (DLTs are described in the Supplementary Methods). Treatment-emergent adverse events (TEAE) were graded according to NCI Common Terminology Criteria for Adverse Events version 5.0, except for cytokine release syndrome (CRS), which was graded according to the American Society for Transplantation and Cellular Therapy Consensus Grading for CRS (20). CRS was classified as an adverse event of special interest and recommendations for management were provided in the protocol, including close monitoring of organ function, provision of supportive care, antipyretics, analgesics, active treatments for hypotension or hypoxia, and subasumstat dose holds, reductions, or discontinuation.

Disease response and progression were assessed for up to 48 months using CT and/or MRI scans with intravenous contrast and ^18^fluorodeoxyglucose-PET imaging according to RECIST v1.1 criteria for solid tumors or Lugano criteria for lymphoma response (21).

Subasumstat PK were evaluated after the first dose on C1D1 and cycle 1 day 8 (C1D8). The parameters included maximum observed plasma concentration (C_max_), time to first occurrence of C_max_, area under the plasma concentration–time curve from time 0 to the last measurable concentration (AUC_0–t_) or from time 0 extrapolated to time infinity (AUC_0–∞_), terminal disposition phase half-life, total clearance, and volume of distribution at steady state. PK parameters were estimated from concentration–time profiles for the PK analysis set, using non-compartmental methods, with Phoenix WinNonlin (RRID: SCR_024504).

Blood samples for subasumstat–SUMO adduct formation and SUMO2/3 inhibition assessments were collected before dose and at 1, 4, and 8 hours after the infusion on C1D1 and C1D8. Blood samples for additional biomarker analyses were collected before dose on C1D1 and C1D8 and at 4, 8, and 24 hours after infusion on C1D1 and C1D8. Skin biopsies (used as surrogate tissue for solid tumors) were collected at screening and on C1D8 at 2 hours (±30 minutes) after end of infusion, and tumor biopsies were collected at screening and cycle 2 day 8 (C2D8; +7 days; whenever possible). In blood samples, a custom flow cytometry assay was utilized for detecting target engagement and SUMOylated protein levels in lymphocytes (including T-, B-, and NK-cell subsets). The assay included a PE-conjugated antibody specifically recognizing the SUMO–subasumstat adduct (MIL-113, anti–cysteinyl-TAK-981, Epitomics; refs. 14, 15, 22) and an AF488-conjugated antibody recognizing SUMO2/3 conjugates (MBL International, cat. #M114-3, RRID: AB_592769). Peripheral blood cells were lysed/fixed, permeabilized (BD Biosciences), and stained with MIL-113 and M114-3 and with antibodies against CD45, CD3, CD19, CD16/CD56, and CD14. IHC assays for SUMO–subasumstat adduct and SUMO2/3 inhibition in skin biopsies and tumors were performed using MIL-113 and M114-3 antibodies. Activation of the IFNI pathway was assessed in peripheral blood by analysis of gene expression data (PanCancer IO 360 panel by NanoString for phase I and whole-transcriptome RNA sequencing data for phase II). Type I IFN gene signature was computed using geometric mean of the following genes: *CCL2*, *CXCL10*, *DDX58*, *IRF7*, *ISG15*, *STAT1*, and *STAT2*. These seven IFNI-regulated genes were selected prior to the trial, based on *in vitro* studies of the effect of subasumstat on human blood from 10 healthy donors analyzed on the NanoString PanCancer IO 360 panel. Plasma chemokines and cytokines were measured using the MesoScale Discovery platform (pro-inflammatory and chemokine panels, BioAgilytix). For assessment of NK- and T-cell activation, cells were suspended in buffer and incubated with the following monoclonal antibodies: APC-H7 mouse anti–human CD45 (BD Biosciences, cat. #560274), APC mouse anti–human CD69 (BD Biosciences, cat. #555533), BV421 mouse anti–human CD56 (BD Biosciences, cat. #562751), mouse anti–human CD16 PerCP-Cy5.5 (BD Biosciences, cat. #338440), BV510 mouse anti–human CD4 (BD Biosciences, cat. #563094), BV605 mouse anti–human CD8 (BD Biosciences, cat. #564116), and Alexa Fluor 700 anti–mouse CD3 antibody (BioLegend, cat. #100215), prior to assessment by flow cytometry using the BD Scientific FACSCanto flow cytometer (RRID: SCR_018055). Tumor PD-L1 expression level was assessed in paired tumor biopsies using the 22C3 PD-L1 IHC assay; combined positive score (CPS) was calculated for baseline and on-treatment tumor samples. The immune landscape in the tumor microenvironment (TME) was assessed using an integrated RNAscope and 27-marker MultiOmyx immunofluorescence panel.

### Statistics and study populations

The DLT-evaluable analysis set included patients who received all cycle 1 doses of subasumstat without experiencing a DLT or who had a DLT during cycle 1 of the study. It was anticipated that approximately 70 DLT-evaluable patients would be enrolled for dose escalation. The safety analysis set included all patients who received at least one dose, even if incomplete, of subasumstat. The tumor response–evaluable set included patients who received at least one dose of subasumstat, had sites of measurable disease at baseline and one post-baseline disease assessment, or discontinued treatment because of symptomatic deterioration or death prior to post-baseline evaluation. The overall response rate (ORR) was defined as the proportion of patients who achieved complete response and partial response (PR) during the study. The disease control rate (DCR) was defined as the proportion of patients who achieved SD for ≥6 weeks as per RECIST v1.1 for solid tumors or Lugano classification for lymphomas. Both ORR and DCR were estimated with two-sided 95% exact binomial confidence intervals (CI). Progression-free survival was summarized descriptively using the Kaplan–Meier method.

## Results

### Patient disposition and baseline characteristics

A total of 109 patients were enrolled: 84 patients in phase I and 25 in phase II (Supplementary Table S1). In phase I, patients received subasumstat across all dosing cohorts (twice weekly: 3, 6, 10, 15, 25, 40, 60, 75, 90, and 120 mg; once weekly: 60, 75, 90, and 120 mg; days 1, 8, and 15: 90 mg). At the data cutoff (February 22, 2024), all patients had discontinued subasumstat, with the most common reason being disease progression [phase I, *n* = 53 (63.1%); phase II, *n* = 15 (60.0%)].

Patient demographics and baseline characteristics are shown in Table 1. In phase I, the median age was 62.5 years, the median time from initial diagnosis was 40.1 months (range, 4–249), and patients had received a median of four prior lines of therapy (range, 1–10). In phase I, 83 patients had solid tumors, and one patient had lymphoma. The most common primary solid tumor disease diagnoses were colorectal cancer (*n* = 21, 25.0%), pancreatic cancer (*n* = 8, 9.6%), NSCLC (*n* = 5, 6.0%), and kidney cancer (*n* = 5, 6.0%).

**Table 1.** Patient demographics and disease characteristics at baseline.

Characteristic, *n* (%), unless otherwise stated	Phase I (*n* = 84)	Phase II (*n* = 25)	
		Solid tumors (*n* = 17)	Lymphoma (*n* = 8)
Median age, years (range)	62.5 (39–80)	61.0 (32–78)	
Female	47 (56.0)	11 (44.0)	
Race	​	​	
White	70 (83.3)	18 (72.0)	
Black/African American	8 (9.5)	1 (4.0)	
Asian	2 (2.4)	4 (16.0)	
Other^a^	4 (4.8)	2 (8.0)	
Median time from initial diagnosis, months (range)	40.1 (4–249)	25.0 (8–67)	5.4 (<1–69)
ECOG performance status	​	​	​
0	25 (29.8)	4 (23.5)	1 (12.5)
1	59 (70.2)	13 (76.5)	7 (87.5)
Initial diagnosis	​	​	​
Solid tumor	83 (98.8)	17 (100)	N/A
Colorectal	21 (25.0)	7 (41.2)	N/A
Pancreatic	8 (9.6)	0	N/A
NSCLC	5 (6.0)	7 (41.2)	N/A
Kidney	5 (6.0)	0	N/A
Cervical	1 (1.2)	3 (17.6)	N/A
Other^b^	43 (51.2)	0	N/A
Lymphoma	1 (1.2)	NA	8 (100)
DLBCL	NA	NA	6 (75.0)
FL	NA	NA	2 (25.0)
Solid tumor disease stage at initial diagnosis	​	​	​
I–II	9 (10.7)	N/A	N/A
III–IV	70 (83.3)	N/A	N/A
Unknown	4 (4.8)	N/A	N/A
Median lines of prior therapy (range)	4 (1–10)	2 (1–7)	
Median time from the last prior systemic therapy, months (range)	1.6 (0.5–122.8)	2.8 (0.5–26.7)	
PD-L1 score at study entry	​	​	​
<1%	N/A	5 (29.4)	N/A
1–<50%	N/A	2 (11.8)	N/A
≥50%	N/A	1 (5.9)	N/A
Unknown/not available	N/A	9 (52.9)	N/A
AJCC anatomic stage at study entry	​	​	​
IIIA	N/A	1 (5.9)	N/A
IVA	N/A	4 (23.5)	N/A
IVB	N/A	7 (41.2)	N/A
IVC	N/A	3 (17.6)	N/A
Unknown	N/A	2 (11.8)	N/A
Ann Arbor staging at study entry	​	​	​
I	N/A	N/A	0
II	N/A	N/A	1 (12.5)
III	N/A	N/A	3 (37.5)
IV	N/A	N/A	4 (50.0)
Extranodal involvement at study entry	N/A	N/A	5 (62.5)
History of bone marrow involvement	N/A	N/A	1 (12.5)

Abbreviations: AJCC, American Joint Committee on Cancer; ECOG, Eastern Cooperative Oncology Group; N/A, not applicable.

a Includes multiple races or race was not reported.

b Melanoma, breast, prostate, and head and neck tumors (*n* = 4 each); adrenal tumor (*n* = 3); endometrial, uterine, soft tissue, bile duct, appendiceal, and peritoneum tumors (*n* = 2 each); and bladder, chordoma, anal, gall bladder, liver, small cell lung cancer, thyroid, salivary glands, biliary, tongue, unknown (small bowel or colon), and vulva tumors (*n* = 1 each).

In phase II, patients received subasumstat at the RP2D of 90 mg twice weekly in the following cohorts: NSCLC (*n* = 7), CPI-naïve cervical cancer (*n* = 3), CPI-naïve MSS colorectal cancer (*n* = 7), relapsed/refractory DLBCL after CAR-T (*n* = 4), relapsed/refractory DLBCL not treated with CAR-T (*n* = 3), and relapsed/refractory stage IV FL (*n* = 1). The median age of patients in phase II was 61.0 years, the median time since initial diagnosis was 25.0 months (range, 8–67) for patients with solid tumors and 5.4 months (range, <1–69) for patients with lymphomas, and the median number of prior therapies was two (range, 1–7).

Representativeness of study participants is detailed in Supplementary Table S2.

### DLTs and determination of the MTD

Most patients in phase I were evaluable for DLTs (*n* = 74 of 84, 88.1%). A total of four patients reported DLTs, all in the twice weekly cohorts: one patient receiving 60 mg subasumstat had a transient grade 3 alanine transaminase/aspartate transaminase elevation, which resolved following dosage reduction to 40 mg twice weekly; one patient receiving 90 mg subasumstat had grade 3 pneumonitis and discontinued study treatment; one patient receiving 120 mg subasumstat had grade 3 stomatitis, which resolved following dose reduction to 90 mg; and a second patient receiving 120 mg subasumstat had grade 3 cognitive disorder (not considered CRS-related) and discontinued study treatment (Supplementary Table S3). The MTD was determined to be 120 mg twice weekly; however, dose-dependent activity across a range of pharmacodynamic measures indicated that subasumstat was biologically active at doses of ≥60 mg and together with the safety data supported an RP2D of 90 mg twice weekly.

### Safety

All patients across both phases received at least one subasumstat dose and were included in the safety population (totaling up to 109). Patients received a median of two treatment cycles with a median duration of treatment of 32 days, ranging from 11 to 156.5 days in phase I and from 12 to 338 days in phase II (Supplementary Table S1). The median overall relative dose intensity was 100% (range, 25–100), with 62.4% of patients receiving a dose intensity of 100%.

TEAEs were reported by 105 patients (phase I, *n* = 81; phase II, *n* = 24); TEAEs that were considered by the investigator to be related to subasumstat were reported in 87 (79.8%) patients (Table 2). The most frequently reported TEAEs were nonhematologic and included fatigue (46.8%), nausea (41.3%), diarrhea (35.8%), pyrexia (35.8%), and headache (33.9%; Table 3). The most common subasumstat-related TEAEs were fatigue, occurring in 31.2% of patients, nausea in 30.3%, and pyrexia in 29.4% (Supplementary Table S4). Overall, grade ≥3 TEAEs were reported in 66 (60.6%) patients, with *n* = 48 in phase I and *n* = 18 in phase II (Table 4). The most common overall grade ≥3 TEAEs were anemia (*n* = 14, 12.8%), hypokalemia (*n* = 7, 6.4%), and abdominal pain (*n* = 6, 5.5%; Table 4). Subasumstat-related grade ≥3 TEAEs were reported in 29 (26.6%) patients, with the most frequent being neutropenia (*n* = 4, 3.7%), anemia (*n* = 4, 3.7%), decreased lymphocyte blood cell count (*n* = 4, 3.7%), and decreased platelet count (*n* = 3, 2.8%; Supplementary Table S5). Discontinuation of subasumstat due to TEAEs occurred in 12 (11.0%) patients, including pneumonitis which occurred in two patients (Table 2). Overall, 12 (11.0%) on-study deaths occurred with none reported as related to subasumstat (Table 2).

**Table 2.** Safety overview.

Event, preferred term *n* (%)	Phase I (*n* = 84)	Phase II (*n* = 25)	Total (*N* = 109)
TEAEs	81 (96.4)	24 (96.0)	105 (96.3)
Related to subasumstat	65 (77.4)	22 (88.0)	87 (79.8)
Grade ≥3 TEAEs	48 (57.1)	18 (72.0)	66 (60.6)
Related to subasumstat	18 (21.4)	11 (44.0)	29 (26.6)
Serious adverse events	37 (44.0)	11 (44.0)	48 (44.0)
Related to subasumstat	7 (8.3)	5 (20.0)	12 (11.0)
AESI	9 (10.7)	3 (12.0)	12 (11.0)
Related to subasumstat	9 (10.7)	3 (12.0)	12 (11.0)
CRS	9 (10.7)	3 (12.0)	12 (11.0)
TEAEs leading to discontinuation	8 (9.5)	4 (16.0)	12 (11.0)
Related to subasumstat	3 (3.6)	1 (4.0)	4 (3.7)
On-study deaths^a^	10 (11.9)	2 (8.0)	12 (11.0)

Abbreviation: AESI, adverse event of special interest.

a No subasumstat-related deaths.

**Table 3.** Most common TEAEs (≥10% of the population) by preferred term.

TEAEs, preferred term *n* (%)	Phase I (*n* = 84)	Phase II (*n* = 25)	Total (*N *= 109)
Fatigue	42 (50.0)	9 (36.0)	51 (46.8)
Nausea	41 (48.8)	4 (16.0)	45 (41.3)
Diarrhea	29 (34.5)	10 (40.0)	39 (35.8)
Pyrexia	28 (33.3)	11 (44.0)	39 (35.8)
Headache	30 (35.7)	7 (28.0)	37 (33.9)
Decreased appetite	26 (31.0)	5 (20.0)	31 (28.4)
Vomiting	27 (32.1)	2 (8.0)	29 (26.6)
Chills	20 (23.8)	8 (32.0)	28 (25.7)
Dyspnea	22 (26.2)	4 (16.0)	26 (23.9)
Abdominal pain	17 (20.2)	4 (16.0)	21 (19.3)
Anemia	12 (14.3)	7 (28.0)	19 (17.4)
Arthralgia	12 (14.3)	5 (20.0)	17 (15.6)
Peripheral edema	15 (17.9)	2 (8.0)	17 (15.6)
Constipation	14 (16.7)	2 (8.0)	16 (14.7)
Back pain	12 (14.3)	2 (8.0)	14 (12.8)
Insomnia	11 (13.1)	2 (8.0)	13 (11.9)
Abdominal distension	11 (13.1)	1 (4.0)	12 (11.0)
CRS	9 (10.7)	3 (12.0)	12 (11.0)
Dehydration	10 (11.9)	2 (8.0)	12 (11.0)
Pleural effusion	9 (10.7)	3 (12.0)	12 (11.0)
Hypophosphatemia	11 (13.1)	0	11 (10.1)
Stomatitis	8 (9.5)	3 (12.0)	11 (10.1)

**Table 4.** Most common grade ≥3 TEAEs (≥1% of the population) by preferred term.

Grade ≥3 TEAEs, preferred term *n* (%)	Phase I (*n* = 84)	Phase II (*n* = 25)	Total (*N* = 109)
Patients with at least one grade ≥3 TEAE	48 (57.1)	18 (72.0)	66 (60.6)
Anemia	9 (10.7)	5 (20.0)	14 (12.8)
Hypokalemia	7 (8.3)	0	7 (6.4)
Abdominal pain	4 (4.8)	2 (8.0)	6 (5.5)
Lymphocyte count decreased	5 (6.0)	0	5 (4.6)
Fatigue	5 (6.0)	0	5 (4.6)
Vomiting	4 (4.8)	0	4 (3.7)
Small intestinal obstruction	4 (4.8)	0	4 (3.7)
Neutropenia	0	4 (16.0)	4 (3.7)
Dyspnea	4 (4.8)	0	4 (3.7)
Acute kidney injury	2 (2.4)	2 (8.0)	4 (3.7)
Platelet count increased	1 (1.2)	2 (8.0)	3 (2.8)
Lymphopenia	1 (1.2)	1 (4.0)	2 (1.8)
Thrombocytopenia	1 (1.2)	1 (4.0)	2 (1.8)
Nausea	2 (2.4)	0	2 (1.8)
Aspartate aminotransferase increased	2 (2.4)	0	2 (1.8)
Blood bilirubin increased	2 (2.4)	0	2 (1.8)
Hypomagnesemia	2 (2.4)	0	2 (1.8)
Hypophosphatemia	2 (2.4)	0	2 (1.8)
Pneumonitis	2 (2.4)	0	2 (1.8)
Pleural effusion	2 (2.4)	0	2 (1.8)
Pyrexia	2 (2.4)	0	2 (1.8)
Pneumonia	1 (1.2)	1 (4.0)	2 (1.8)
Hyperbilirubinemia	0	2 (8.0)	2 (1.8)
Muscular weakness	0	2 (8.0)	2 (1.8)
Hypertension	1 (1.2)	1 (4.0)	2 (1.8)

Infusion-related reactions (IRR) were reported in seven patients (6.4%), all of which were grade 1 or 2, and deemed related to subasumstat in six patients. CRS was an adverse event of special interest and was reported in 12 patients (11.0%) and was observed starting at doses of 60 mg or higher; all incidences were related to subasumstat and were grade 1 or 2 in severity, with most being nonserious.

### PK

**Figure 2.**
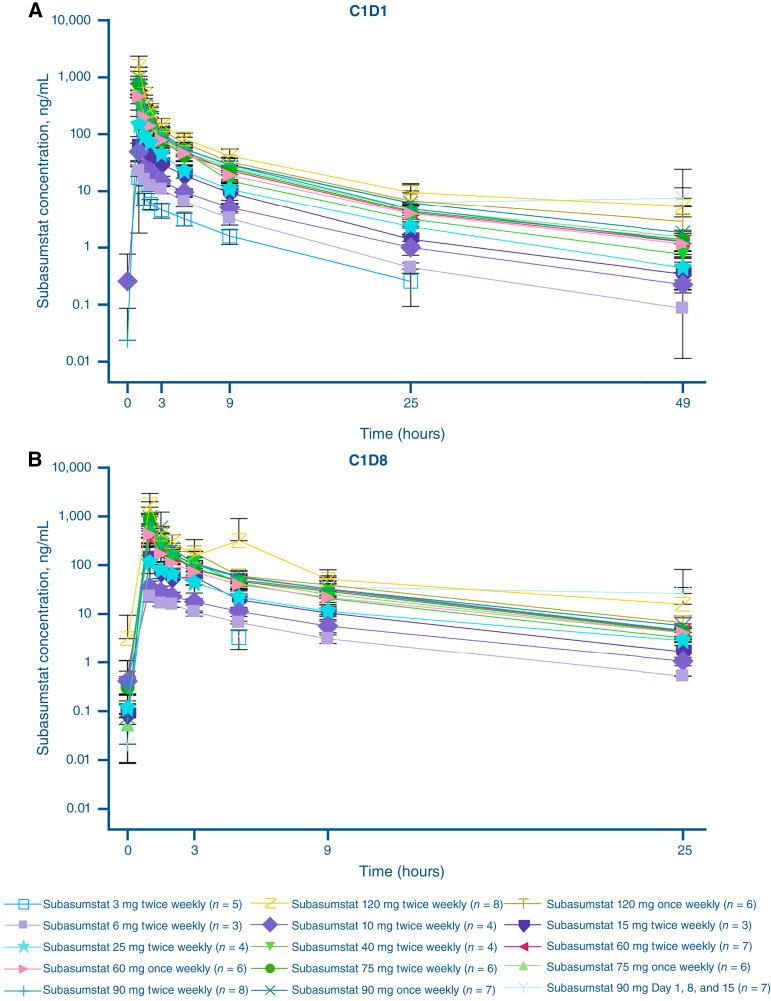
Mean plasma concentration vs. time profiles of subasumstat following intravenous infusions – phase I. **A,** C1D1. **B,** C1D8. Semi-logarithmic scale. Time points were relative to the start of subasumstat infusion.

A total of 84 patients were included in the PK analysis set for phase I. Maximum plasma and blood concentrations of subasumstat were attained at the end of infusion approximately 1-hour after dose following intravenous dosing on C1D1 and C1D8. Peak exposure (C_max_) and total exposure (AUC_0–t_ and AUC_0–∞_) increased in an approximately dose-proportional manner over the dose range of 3 to 120 mg, on C1D1 and C1D8 (Fig. 2). Across all dose levels, plasma and blood concentrations of subasumstat declined, with terminal disposition phase half-life ranging from 3.35 to 9.31 hours and from 2.87 to 9.19 hours, respectively. Clearance and volume of distribution at steady state were lower in blood compared with plasma, ranging from 2.29 L/hour to 4.56 L/hour and between 13.7 and 38.8 L, respectively.

In phase II, following multiple intravenous administrations of subasumstat 90 mg twice weekly, the plasma concentrations indicated no apparent accumulation of subasumstat on cycle 1 day 4, C1D8, cycle 2 day 1, or C2D8 when compared with C1D1 (Supplementary Fig. S2).

### Pharmacodynamics

**Figure 3.**
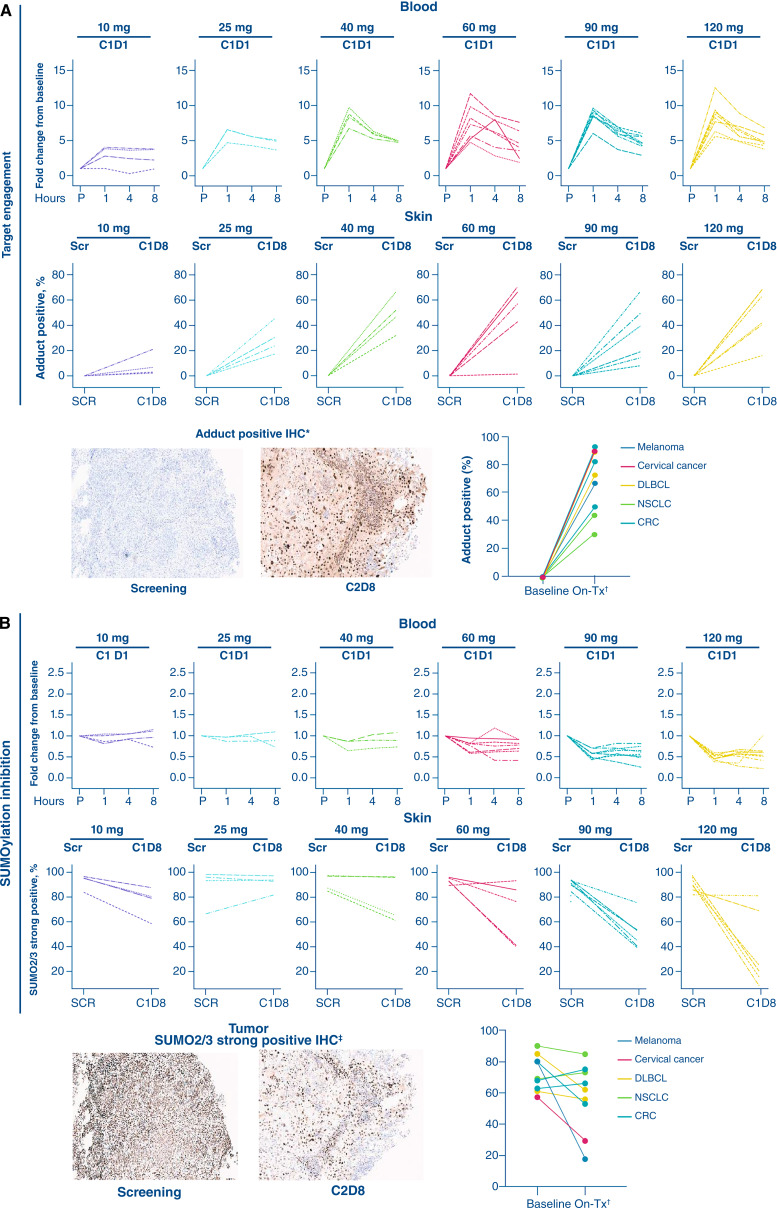
Target engagement and SUMO pathway inhibition in peripheral blood lymphocytes, skin, and tumor biopsies – phase I (pharmacodynamic population). **A,** Subasumstat–SUMO target engagement. Quantification of adduct formation in peripheral blood lymphocytes from selected dose levels (flow cytometry analysis, fold-change MESF from pre-dose C1D1; lines represent individual patients), in skin (IHC staining; findings represent a mean of two replicates across all doses), and in paired tumor samples collected at screening and on treatment (representative IHC in a cervical cancer biopsy sample following treatment with subasumstat 90 mg twice weekly). **B,** SUMO2/3 inhibition. Quantification of SUMO2/3 inhibition in peripheral blood lymphocytes from selected dose levels (flow cytometry analysis, fold-change MESF from pre-dose C1D1; lines represent individual patients), in skin samples (IHC staining; findings represent a mean of three replicates), and in paired tumor samples collected at screening and on treatment (representative IHC in a cervical cancer biopsy sample following treatment with subasumstat 90 mg twice weekly). *, Tumor engagement observed in all tumor samples; ^†^, Tumor samples from phase II 90 mg twice weekly C2D8 for cervical cancer, cycle 2 day 9 or cycle 2 day 14 for NSCLC, and cycle 2 day 10 or cycle 2 day 15 for colorectal cancer, and phase I 25 mg twice weekly on C1D8 for melanoma; ^‡^, SUMO2/3 inhibition observed in some tumor samples. CRC, colorectal cancer; MESF, molecules of equivalent soluble fluorochrome; P, predose; SCR, screening; SUMO, small ubiquitin-like modifier; Tx, treatment.

At dose levels of ≥10 mg, target engagement in peripheral blood lymphocytes, as evidenced by SUMO–subasumstat adduct formation, was detected within 1 hour of the end of subasumstat infusion on C1D1 and C1D8, and it persisted for at least 8 hours across both twice weekly and once weekly dosing schedules in phase I (Fig. 3A for selected twice daily doses at C1D1; Supplementary Fig. S3A for selected twice daily doses at C1D1 and C1D8 and selected once daily doses at C1D1 and C1D8). Target engagement and SUMO2/3 inhibition were evaluated in tumor samples collected at screening and C2D8 from eight patients in phase II who received subasumstat 90 mg twice weekly (MSS colorectal cancer, *n* = 3; non-squamous NSCLC, *n* = 2; DLBCL, *n* = 2; and cervical cancer, *n* = 1) and at screening and C1D8 from one patient in phase I with melanoma who received subasumstat 25 mg twice weekly. All evaluated tumor samples demonstrated robust target engagement following subasumstat administration; SUMO2/3 inhibition was observed in most post-dose samples, except for two patients with MSS colorectal cancer and one patient with NSCLC (Fig. 3A and B). Target engagement was also confirmed in skin biopsies taken 2 hours (±30 minutes) after the C1D8 subasumstat dose, with adduct formation observed from subasumstat doses ≥10 mg (Fig. 3A; Supplementary Fig. S3B). Dose-dependent SUMO2/3 inhibition was also observed in peripheral blood lymphocytes (at ≥40 mg doses) within 1 hour of the end of the subasumstat infusion and was sustained for at least 8 hours (Fig. 3B for selected twice weekly doses at C1D1; Supplementary Fig. S4A for selected twice weekly doses at C1D1 and C1D8 and selected once weekly doses at C1D1 and C1D8). Reduced levels of SUMO2/3 were also observed in skin biopsies taken on C1D8 at doses ≥60 mg (Fig. 3B; Supplementary Fig. S4B).

**Figure 4.**
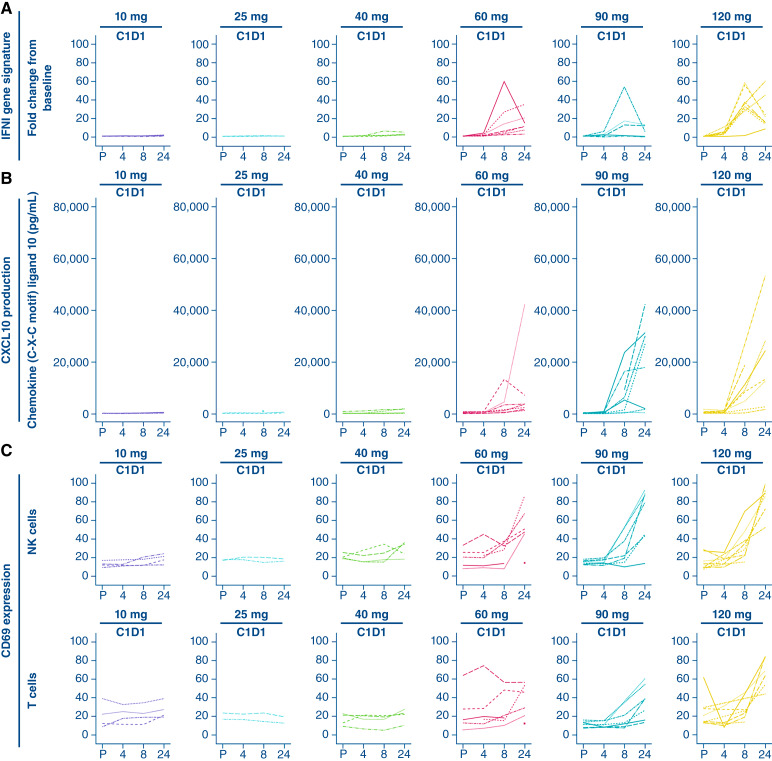
Activation of IFN1 signaling pathway – phase I (pharmacodynamic population). **A,** IFNI gene signature in peripheral blood at selected doses. **B,** CXCL-10 levels in plasma at selected doses. **C,** Percentage of CD69-positive cells in NK and CD8^+^ T cells (CD3^−^CD56^+^/CD16^+^CD69^+^ %) at selected doses. Lines represent individual patients. P, pre-dose.

Dose-dependent upregulation of the IFNI gene signature in peripheral blood and the plasma cytokine IFNγ-induced chemokine ligand 10 (CXCL-10; IP-10) was observed at doses ≥40 mg with both dosing schedules in phase I. The IFNI gene signature peak occurred 8 to 24 hours after the end of subasumstat infusion, and IP-10 plasma concentration increased within 24 hours after the end of subasumstat infusion. Both markers returned to baseline levels before dose on C1D8 (Fig. 4A and B for selected twice weekly doses at C1D1). Data for additional time points and dosing schedules are shown in Supplementary Figs. S5 and S6. Finally, increases in the percentage of CD69^+^ cells within NK-cell and T-cell populations in peripheral blood were observed at subasumstat dose levels ≥60 mg across both dose schedules and study phases, with peak effect 24 hours after subasumstat infusion on C1D1 and C1D8 (Fig. 4C for selected twice weekly doses at C1D1; Supplementary Fig. S6 for selected twice weekly and selected once weekly doses at C1D1 and C1D8), consistent with innate and adaptive immune cell activation. Phase II dose-expansion pharmacodynamic data are detailed in Supplementary Fig. S7.

The impact of SUMOylation inhibition on the immune landscape within the TME was evaluated via multiplex immunofluorescence. This analysis showed significant CD8^+^ and CD4^+^ T-cell infiltration after subasumstat administration in samples from CPI-naïve (cervical cancer) and CPI-exposed (melanoma and NSCLC) patients; no significant changes in regulatory T cells were observed (Supplementary Fig. S8). Conversely, biopsies obtained from three patients with MSS colorectal cancer did not exhibit an increase in T-cell density. Tumor biopsies obtained from patients with cervical cancer and melanoma demonstrated additional TME changes after subasumstat administration. A significant increase in T-cell activation was seen, as detected by an increase in CD69 expression on CD4^+^ and CD8^+^ T cells, as well as a marked increase in human leukocyte antigen (HLA) class I expression on tumor cells, consistent with increased antigen presentation and susceptibility to immune-mediated killing. There was also an increase in density of activated CD11C^+^HLA-DR+ myeloid dendritic cells. PD-L1 CPS increased in most paired tumor samples following subasumstat administration at C2D8 (Supplementary Fig. S9).

### Clinical activity

Among 99 response-evaluable patients across both phases, three patients achieved a confirmed PR: one patient with breast cancer who received seven cycles of subasumstat 40 mg twice weekly, one patient with head and neck cancer who received six cycles of subasumstat 120 mg twice weekly and five cycles of subasumstat 90 mg twice weekly, and one patient with FL enrolled in the expansion cohort who received 15 cycles of subasumstat 90 mg twice weekly. Overall, 26 (26.3%) patients had a best response of SD. In the NSCLC and cervical cancer expansion cohorts enrolled in phase II, four of seven and two of three patients had a best response of SD, with a median progression-free survival of 6.06 and 3.78 months, respectively. A swimmer plot of individual patient responses is shown in Supplementary Fig. S10. The DCR (defined as the proportion of patients who achieved a best response of SD for ≥6 weeks or better) was 28% (*n* = 21/75; 95% CI, 18.24–39.56) in phase I and 33% (*n* = 8/24; 95% CI, 15.63–55.32) in phase II. Overall, the DCR was 29.3% (*n* = 29/99; 95% CI, 20.57–39.29). Because of the limited clinical benefit observed, none of the phase II cohorts expanded to stage 2.

## Discussion

The discovery and development of SUMOylation inhibitors have been motivated by evidence that dysregulation of the SUMOylation pathway is implicated in the development of cancers (4–6, 8, 13) and by the elucidation of the role of SUMOylation and its inhibition in the immune system, which can be leveraged to enhance antitumor immunity (2, 15). Subasumstat (TAK-981) is a first-in-class small-molecule inhibitor of SAE (14) and is the only SUMOylation inhibitor thus far to enter clinical development. Clinical development was based on promising preclinical data that showed that treatment with subasumstat promoted antitumor immune responses through activation of IFNI signaling (15). Here, we report the first-in-human results from the single-agent phase I/II study, which demonstrated that subasumstat had a manageable safety profile, with robust evidence of target engagement and preliminary evidence of disease control in a heavily pretreated patient population with advanced/metastatic solid tumors and relapsed/refractory hematologic malignancies.

Overall, subasumstat could be safely administered across a wide range of dose levels (3–120 mg) and on both biweekly and weekly schedules. The MTD was determined to be 120 mg twice weekly. Consistent with the mechanism of action of subasumstat and the induction of IFNI signaling in immune cells observed in the preclinical studies (15–17), the most frequent TEAEs reported included flu-like symptoms such as fatigue, pyrexia, and headache, and gastrointestinal toxicities including nausea and diarrhea. These adverse events were mainly low grade; anemia (13%) was the only grade ≥3 TEAE reported in more than 10% of patients. Adverse events were manageable with dose modifications, with only 12 (11%) patients discontinuing subasumstat treatment due to TEAEs. Because of the immune-activating properties of subasumstat and the release of cytokines, patients were closely monitored for signs of CRS and IRR, and management strategies for these adverse events were provided in the protocol. The rates of CRS (11%) and IRR (6%) were low overall and were mainly low grade and manageable, with no patients discontinuing due to these TEAEs.

Subasumstat dosing on a twice-weekly schedule was feasible, with a median relative dose intensity of 100% (range, 25–100). This schedule was selected for investigation in phase II, and PK results indicated no accumulation from C1D1 to C1D8, as well as no apparent accumulation in cycle 2 after repeat dosing; however, this 1-hour intravenous infusion on days 1, 4, 8, and 11 of a 21-day cycle could be challenging to implement in clinical practice, especially if administered in combinations.

Pharmacodynamic analyses confirmed the mechanism of action of subasumstat demonstrating target engagement with subasumstat–SUMO adduct formation and SUMO2/3 inhibition in peripheral blood lymphocytes, skin biopsies, and paired tumor samples. Target engagement was rapid, observed within 1 hour of subasumstat dosing, and robust, persisting for at least 8 hours after subasumstat dosing. Target engagement was followed by induction of an IFNI response, with upregulation of the IFNI-regulated gene signature in blood samples and plasma levels of the chemokine IP-10 peaking between 8 and 24 hours after subasumstat dosing. This was followed by activation of peripheral NK cells and T cells 24 hours after subasumstat dosing, confirming an impact on innate and adaptive immune responses in patients with advanced/metastatic solid tumors and relapsed/refractory hematologic malignancies. Despite the MTD of 120 mg, the pharmacodynamic activity was observed at subasumstat doses ≥60 mg. The safety data supported an RP2D of 90 mg twice weekly, which was implemented in the phase II dose-expansion part of the study.

The impact of subasumstat on the TME and PD-L1 CPS was assessed in a limited number of paired tumor biopsies and demonstrated that it can potentially enhance IFN production and promote an inflammatory TME. Similar effects were observed in a phase 0 study that investigated the *in situ* effects of subasumstat via trackable intratumor microdosing combined with spatial profiling in patients with head and neck cancer, in whom subasumstat was shown to modulate the TME from an immune-suppressive to an immune-permissive status through stimulation of the IFN pathway (22). In the present study, paired biopsies from patients with cervical cancer, melanoma, and NSCLC (one each) showed increased T-cell density. However, T-cell density did not increase in any of the three paired biopsies from patients with MSS colorectal cancer, suggesting that these immune-evasive tumors are less responsive to immune-mediating effects of subasumstat, highlighting variability in TME responses.

Overall, three (3%) patients achieved a confirmed PR, and 26 patients (26.3%) had SD with single-agent subasumstat. All responses were observed with the twice weekly schedule at doses of 40 mg (patient with breast tumor, phase I), 90 mg (patient with FL, phase II), and 120 mg (patient with head and neck tumor, phase I). The patient population in this study was heavily pretreated, and 23 patients in phase I received doses of subasumstat that were less than the pharmacologically active dose of 60 mg. The low response rate observed with the RP2D in the expansion phase, despite the evidence of target engagement, leaves open the possibility that subasumstat may be more effective in combinations with synergistic mechanisms of action, although unidentified resistance mechanisms or pseudo-progression due to influx of T cells cannot be excluded.

In parallel to the present single-agent study, subasumstat was investigated in a phase Ib/II study in combination with the anti-CD20 monoclonal antibody rituximab in 24 patients with relapsed/refractory NHL (NCT04074330) and demonstrated an ORR of 29% (19). This supported preclinical findings that subasumstat could activate NK cells and macrophages via IFNI signaling, leading to enhanced antibody-dependent cellular cytotoxicity and cellular phagocytosis–mediated tumor cell killing in the presence of rituximab (19). Subasumstat has also been investigated in combination with the CPI pembrolizumab in a phase Ib/II study (NCT04381650), with preliminary antitumor activity observed in patients with relapsed/refractory MSS colorectal cancer and CPI-exposed non-squamous NSCLC from the dose-escalation cohort (18). However, no activity was observed in the MSS colorectal cancer dose-expansion cohort and the study was discontinued early in this patient population because of futility. These clinical data indicate that the induction of IFN1 signaling and effector cell regulation may help overcome tumor resistance to CPIs observed in some patients only. These studies of subasumstat as single agent and in combination with established immunotherapies represent the first clinical trials assessing SUMOylation inhibitors. Taken together, these trials provide valuable insights on the safety, tolerability, PK, and pharmacodynamics of subasumstat alone and in combination, but further investigation remains necessary to identify the adequate settings to achieve clinical activity.

Another proposed SUMOylation inhibitor, SB-4826, has shown promising preclinical and *in vivo* antitumor activity but has not entered clinical trials to date. Notably, SB-4826 displayed near-complete tumor growth inhibition in a mouse CT-26 colorectal tumor model (known to be non-responsive to PD-1 treatment) as monotherapy and up to 60% complete response when combined with anti–PD-1 (23). Both SB-4826 and subasumstat are SAE inhibitors, but although subasumstat disrupts SAE activity by binding in the catalytic site and forming an adduct with a SUMO protein, SB-4826 covalently binds to an allosteric pocket of SAE, which might result in some differences in activity or tolerability between the two compounds. Although subasumstat is no longer under clinical development by the sponsor, this decision was not motivated by changes in safety events or new safety concerns associated with subasumstat. The overall manageable safety profile reported with subasumstat as single agent and in combination regimens, along with the established proof of concept, should encourage further research into SUMOylation inhibitors, particular as combination partners for drugs with synergistic mechanisms of action.

### Conclusions

This first-in-human study of single-agent subasumstat in patients with advanced/metastatic solid tumors and relapsed/refractory hematologic malignancies demonstrated strong evidence of target engagement, decreased SUMOylation in blood, skin, and tumor, and induction of IFNI signaling with activation of innate and adaptive immune cells in line with the expected mechanism of action of SUMOylation inhibition. Further studies would be needed to understand the role of subasumstat in the treatment of cancer and whether the limited activity observed in this early-phase study can be improved by combining with CPIs or other immune-oncology agents.

## Supplementary Material

Supplementary Materials & Methods Supplementary results

Supplementary Table 1 Supplementary Table 1. Patient disposition and median duration of treatment (days) by subasumstat cohorts in phase I and II (safety population)

Supplementary Table 2 Supplementary Table 2. Representativeness of study participants.

Supplementary Table 3 Supplementary Table 3. Dose limiting toxicities with single agent subasumstat.

Supplementary Table 4 Supplementary Table 4. Subasumstat-related TEAEs occurring in ≥5% of all patients.

Supplementary Table 5 Supplementary Table 5. Grade ≥3 subasumstat-related TEAEs occurring in ≥1% of all patients.

Supplementary Figure 1 Supplementary Figure 1. Subasumstat mechanism of action.

Supplementary Figure 2 Supplementary Figure 2. Mean plasma concentration over time profiles of subasumstat – phase II.

Supplementary Figure 3 Supplementary Figure 3. Subasumstat-SUMO adduct formation in peripheral blood lymphocytes.

Supplementary Figure 4 Supplementary Figure 4. SUMO2/3 inhibition in peripheral blood lymphocytes.

Supplementary Figure 5 Supplementary Figure 5. IFN-1 gene signature in peripheral blood lymphocytes.

Supplementary Figure 6 Supplementary Figure 6. Percentage of CD69-postive cells in NK cells.

Supplementary Figure 7 Supplementary Figure 7. Activation of IFN-I pathway following subasumstat 90 mg BIW administration – phase II.

Supplementary Figure 8 Supplementary Figure 8. Multiplex immunofluorescence analysis of tumor biopsies at screening and following subasumstat administration.

Supplementary Figure 9 Supplementary Figure 9. Effect of subasumstat on TME and PD-L1 expression in tumor biopsies.

Supplementary Figure 10 Supplementary Figure 10. Swimmer plot of patient responses.

## Data Availability

The datasets, including the redacted study protocols, redacted statistical analysis plans, and individual participant data supporting the results of the completed study, will be made available within 3 months from initial request to researchers who provide a methodologically sound proposal. The data will be provided after its de-identification, in compliance with applicable privacy laws, data protection, and requirements for consent and anonymization.
