# Supplementary material for: A First-In-Human Study of the SUMOylation Inhibitor Subasumstat in Patients with Advanced/Metastatic Solid Tumors or Relapsed/Refractory Hematologic Malignancies
Source: Cancer Res Commun. 2025 Nov 19;5(11):2025–38. doi: 10.1158/2767-9764.CRC-25-0243 (PMC12627933; doi:10.1158/2767-9764.CRC-25-0243)
Supplement: Supplementary Materials & Methods — Supplementary results [file crc-25-0243_supplementary_materials__methods_suppsmm.pdf]

Supplementary Material

A First-In-Human Study of the SUMOylation Inhibitor Subasumstat In Patients With Advanced/Metastatic Solid Tumors or Relapsed/Refractory Hematologic Malignancies

Dejan Juric, Daniel Anderson, Afshin Dowlati, Jordi Rodon, Iwona Lugowska, Sławomir Mańdziuk, Yuqin Song, Feng Jung Sherida H. Woei-A-Jin, Marc André, Joanna Góra Tybor, José Ángel Hernández Rivas, Razelle Kurzrock, Armando López-Guillermo, David Schröder, Rafal Stec, Allison Berger, Bo Chao, Aleksander Chudnovsky, John P. Gibbs, Tao Long, Dina Stroopinsky, Qi Dong, and Anthony J. Olszanski

Supplemental methods

Inclusion Criteria

Each patient was required to meet all the following inclusion criteria to be enrolled in the study:

1. Adult male or female patients ≥18 years old.
2. Eastern Cooperative Oncology Group Performance Status (ECOG PS) of 0 to 1.
3. Patient population for phase I dose escalation:
   a) Have a histologically or cytologically confirmed advanced (local regionally recurrent not amenable to curative therapy) or metastatic solid tumors who have no standard therapeutic option with a proven clinical benefit, are intolerant, or have refused them, *OR*
   b) Have a relapsed/refractory lymphoma not amenable to therapies with proven clinical benefit or who are intolerant or who refuse them. Patients with low-grade lymphomas such as follicular lymphoma (FL), small lymphocytic lymphoma, lymphoplasmacytoid lymphoma, and marginal zone lymphomas may not need to exhaust all available therapy. These patients can be enrolled after failure of at least two prior systemic therapies, provided that there is not an immediate need for cytoreduction. In these cases, patients who need immediate therapy for tumor bulk are not eligible for this study.
4. Patient population for phase II dose expansion cohorts:
   Have a histologically or cytologically documented, advanced (metastatic and/or unresectable) cancer as listed below, that is incurable and for which prior standard first-line treatment has failed.
   Note: Prior neoadjuvant or adjuvant therapy included in initial treatment may not be considered first- or later-line standard-of-care treatment unless such treatments were completed <12 months before the current tumor recurrence.
      a) Non-squamous non-small cell lung cancer (NSCLC) that has progressed following one prior systemic immune checkpoint inhibitor (CPI)/anti-programmed cell death ligand-1 (PD-1/L1)-containing therapy and ≤2 lines of therapy. Patients must have not shown evidence of tumor progression during the first 5 months of treatment with first-line CPI/anti-PD-(1/L1)-containing therapy (cohort A).

Note: Patients with known driver mutations/genomic aberrations (e.g., epidermal growth factor receptor, B-Raf proto-oncogene mutation V600E, and ROS proto-oncogene 1 sensitizing mutations, neurotrophic receptor tyrosine kinase gene fusions, and anaplastic lymphoma kinase rearrangements) must have also shown progressive disease after treatment with a commercially available targeted therapy.

b) CPI-naïve cervical cancer (squamous cell carcinoma, adenosquamous carcinoma or adenocarcinoma of the cervix) patients who have received ≤1 prior systemic line of therapy for recurrent or Stage IVB cervical cancer (cohort B).

   Note: The following cervical tumors are not eligible: minimal deviation/adenoma malignum, gastric-type adenocarcinoma, clear-cell carcinoma, and mesonephric carcinoma. Histologic confirmation of the original primary tumor is required via pathology report.

   Note: First-line treatment must have consisted of platinum-containing doublet. Chemotherapy administered concurrently with primary radiation (e.g., weekly cisplatin) is not counted as a systemic chemotherapy regimen.

c) CPI-naïve microsatellite-stable colorectal cancer patients who have progressed on ≤3 chemotherapy regimens (cohort C).

   Note: Patients must have received prior treatment with fluoropyrimidine-, oxaliplatin-, and irinotecan-containing regimens if indicated.

d) Relapsed/refractory diffuse large B cell lymphoma (DLBCL) progressed or relapsed after prior chimeric antigen receptor T cell therapy that has received approval by a health authority for the treatment of DLBCL (cohort D).

e) Relapsed/refractory DLBCL that has progressed or relapsed after at least two but no more than three prior lines of systemic therapy and has not received prior cellular therapy. At least one prior line of therapy must have included a CD20-targeted therapy (cohort E).

f) Relapsed/refractory FL that has progressed or relapsed after at least two but no more than three prior lines of systemic therapy. At least one prior line of therapy must have included a CD20-targeted therapy (cohort F).

5. In phase II only, have at least one radiologically measurable lesion based on Response Evaluation Criteria in Solid Tumors Version 1.1 for patients with solid tumors or Lugano criteria for lymphoma. Tumor lesions situated in a previously irradiated area are considered measurable if progression has been demonstrated in such lesions.
   Note: In phase II stage I, have an additional lesion for pretreatment and on-treatment biopsy.
6. In phase II stage I, willing to consent to mandatory pretreatment and on-treatment tumor biopsy.
   Note: For fresh tumor biopsies, the lesion must be accessible for a biopsy procedure as assessed by the investigator.
7. Is willing to provide archival tumor tissue sample, if available.
8. Adequate bone marrow reserve and renal and hepatic function based on the following laboratory parameters:
   a) Absolute neutrophil count ≥1.0 × 109/L, hemoglobin ≥85 g/L (red blood cell transfusion allowed ≥14 days before assessment), and platelet count ≥75.0 × 109/L (platelet count ≥50.0 × 109/L is allowed for patients with lymphoma if it is clearly due to marrow involvement with no evidence of myelodysplastic syndrome or hypoplastic bone marrow, if found).
   b) Total bilirubin ≤1.5 times the institutional upper limit of normal (ULN); or total bilirubin <3.0 times the ULN with direct bilirubin within normal range in patients with well documented Gilbert's syndrome.
   c) Serum alanine aminotransferase (ALT) or aspartate aminotransferase (AST) ≤3.0 times the ULN (<5 times the ULN if liver enzyme elevations are due to liver metastases).
   d) Estimated creatinine clearance using the Cockcroft-Gault formula ≥45 mL/minute.
9. Recovered to grade 1 or baseline or established as sequelae from all toxic effects of previous therapy (except alopecia, neuropathy, or autoimmune endocrinopathies with stable endocrine replacement therapy, or bone marrow parameters [any of grade 1/2 permitted if directly related to bone marrow involvement]).
10. Consented to undergo serial skin punch biopsies (dose escalation only).

11. Voluntary written consent must be given before performance of any study-related procedure not part of standard medical care, with the understanding that consent may be withdrawn by the patient at any time without prejudice to future medical care.
12. Suitable venous access for safe drug administration and the study-required pharmacokinetic and pharmacodynamic sampling.
13. Women of childbearing potential participating in this study should avoid becoming pregnant, and male patients should avoid impregnating a female partner. Non sterilized female patients of reproductive age and male patients should use effective methods of contraception through defined periods during and after study treatment as specified below. Female patients must meet one of the following:
    a) Postmenopausal for at least 1 year before the screening visit, or
    b) Surgically sterile, or
    c) If they are of childbearing potential, agree to practice one highly effective method and one additional effective (barrier) method of contraception at the same time, from the time of signing of the informed consent form through 6 months after the last dose of study drug, or
    d) Agree to practice true abstinence, when this is in line with the preferred and usual lifestyle of the patient. (Periodic abstinence [e.g., calendar, ovulation, symptothermal, post-ovulation methods], withdrawal, spermicides only, and lactational amenorrhea are not acceptable methods of contraception. Female and male condoms should not be used together.)
14. Male patients, even if surgically sterilized (i.e., status post vasectomy) must agree to one of the following:
    a) Agree to practice effective barrier contraception during the entire study treatment period and through 6 months after the last dose of study drug, or
    b) Agree to practice true abstinence, when this is in line with the preferred and usual lifestyle of the patient. (Periodic abstinence [e.g., calendar, ovulation, symptothermal, post-ovulation methods], withdrawal, spermicides only, and lactational amenorrhea are not acceptable methods of contraception. Female and male condoms should not be used together.)

Exclusion Criteria

Patients meeting any of the following exclusion criteria were not enrolled in the study:

1. Phase I dose escalation and phase II cancer treatment expansion cohorts:
   a) Have received treatment with systemic anticancer treatments or investigational products within 14 days before the first dose of study drug or 5 half-lives, whichever is shorter.
   Note: Low-dose steroids (oral prednisone or equivalent ≤20 mg per day), hormonal therapy for prostate cancer or breast cancer (as adjuvant treatment), and treatment with bisphosphonates and receptor activator of nuclear factor kappa-B ligand inhibitors are allowed.
   b) Have received extended field radiotherapy ≤4 weeks before the start of treatment (≤2 weeks for limited field radiation for palliation), and who has not recovered to grade 1 or baseline from related side effects of such therapy (except for alopecia).
2. Have a history of uncontrolled brain metastasis. Patients with brain metastases are allowed if they are previously treated with surgery, whole-brain radiation, or stereotactic radiosurgery and the patients are receiving a corticosteroid dose ≤10 mg/day of prednisone equivalent at the time of receiving the first dose of subasumstat. For asymptomatic patients, screening brain imaging is not required.
3. Patient is receiving any live vaccine (e.g., varicella, pneumococcus) within 4 weeks of initiation of study treatment.
4. History of any of the following ≤6 months before first dose: congestive heart failure New York Heart Association grade III or IV, unstable angina, myocardial infarction, unstable symptomatic ischemic heart disease, severe noncompensated hypertension despite appropriate medical therapy, ongoing symptomatic cardiac arrhythmias of >grade 2, pulmonary embolism, or symptomatic cerebrovascular events, or any other serious cardiac condition (e.g., pericardial effusion or restrictive cardiomyopathy). Chronic atrial fibrillation on stable anticoagulant therapy is allowed.

5. Baseline prolongation of the QT interval with Fridericia correction method (QTcF) (eg, repeated demonstration of QTcF interval >480 ms, history of congenital long QT syndrome, or torsades de pointes).
6. Psychiatric illness/social circumstances that would limit compliance with study requirements and substantially increase the risk of adverse events (AEs) or has compromised ability to provide written informed consent.
7. Admission or evidence of illicit drug use, drug abuse, or alcohol abuse.
8. History of autoimmune disease requiring systemic immunosuppressive therapy with daily doses of prednisone >10 mg/day or equivalent doses, or any other form of immunosuppressive therapy. Hormone therapy (e.g., thyroxine, insulin, or physiologic corticosteroid replacement therapy for adrenal or pituitary insufficiency) is not considered an excluded form of systemic treatment of an autoimmune disease.
9. History of immune-related AEs related to treatment with immune CPTs that required treatment discontinuation.
10. History of noninfectious pneumonitis that required steroids or a history of interstitial lung disease.
11. Has evidence of active, noninfectious pneumonitis.
12. Have a significant active infection.
13. Known history of HIV infection or any other relevant congenital or acquired immunodeficiency.
14. Known hepatitis B virus surface antigen seropositive or detectable hepatitis C infection viral load.

    Note: Patients who have positive hepatitis B core antibody or hepatitis B surface antigen antibody can be enrolled but must have an undetectable hepatitis B viral load.
15. Receiving or requiring the continued use of medications that are known to be strong or moderate inhibitors and inducers of cytochrome P450 (CYP) 3A4/5 (Appendix E of Protocol Amendment 7) or are strong P-glycoprotein (P-gp) inhibitors (Appendix F of Protocol Amendment 7). To participate in this study, patients should discontinue use of such agents for at least 2 weeks (1 week for CYP3A4/5 and P-gp inhibitors) before receiving a dose of subasumstat.
16. Patient requires the use of drugs known to prolong QTc interval (during phase 1 only) (Appendix H of Protocol Amendment 7).

17. History of allogeneic tissue or solid organ transplant.
18. Second malignancy within the previous 3 years, except treated basal cell or localized squamous skin carcinomas, localized prostate cancer, cervical carcinoma in situ, resected colorectal adenomatous polyps, breast cancer in situ, or other malignancy for which the patient is not on active anticancer therapy.
19. Female patients who are lactating and breastfeeding or have a positive serum pregnancy test during the screening period or a positive urine pregnancy test on day 1 before first dose of study drug.

Definition of dose-limiting toxicities (DLTs) for phase I only

DLTs were defined as any AEs occurring in cycle 1 (unless considered unrelated to the study drug) with the following characteristics:

1. Any grade 5 AE.
2. Nonfebrile grade 4 neutropenia (absolute neutrophil count [ANC] <0.5 × 109/L) lasting ≥7 consecutive days. If myeloid growth factors are used, the event will be considered as DLT irrespective of the duration.
3. Febrile neutropenia: grade ≥3 neutropenia (ANC <1 × 109/L) with fever and/or infection, where fever is defined as a single temperature >38.3°C or sustained temperature of ≥38°C for ≥1 hour.
4. Grade 4 thrombocytopenia lasting ≥7 consecutive days. A platelet count <10 × 109/L at any time is a DLT.
5. Grade 3 thrombocytopenia lasting longer than 14 days or accompanied by grade 2 bleeding or requiring transfusion.
6. Delay in the initiation of cycle 2 by ≥14 days due to a lack of adequate recovery of treatment-related hematological or nonhematologic toxicities.
7. Grade ≥3 nonhematologic toxicity with the following exceptions:
    - Grade 3 arthralgia/myalgia that responds to nonsteroidal anti-inflammatory drugs within 1 week.
    - Grade 3 fatigue lasting fewer than 7 days.
    - Grade 3 endocrine disorder that is managed with or without therapy and the patient is asymptomatic.
    - Grade 3 or 4 inflammatory reaction attributed to a local antitumor response.

- Grade 3 infusion-related actions that resolves within 6 hours with appropriate clinical management.
- Grade 3 cytokine release syndrome that resolves to grade ≤1 in less than 7 days without end-organ damage.
- Asymptomatic laboratory changes (other than renal and hepatic laboratory values) that can be successfully supplemented (reversion of grade 4 events to grade ≤2, reversion of grade 3 events to grade ≤1 or baseline) within 72 hours.
- Grade 3 elevation in ALT, AST, and/or alkaline phosphatase that resolves to grade ≤2 or baseline with supportive care within 7 days and is not associated with other clinically relevant consequences.
- Grade 3 nausea and/or emesis that can be controlled to grade <3 in ≤3 days with the use of optimal antiemetics (defined as an antiemetic regimen that employs both a 5-hydroxytryptamine 3 serotonin receptor antagonist and a corticosteroid given in standard doses and according to standard schedules).
- Grade 3 diarrhea that can be controlled to grade <3 in ≤3 days with appropriate treatment.
- Grade 3 rash lasting ≤7 days after treatment that includes topical steroid treatment, oral antihistamines, and pulse oral steroids (if necessary).

8. Grade 2 nonhematologic toxicities that are considered by the investigator to be related to study drug and dose-limiting.
