# Supplementary material for: A First-In-Human Study of the SUMOylation Inhibitor Subasumstat in Patients with Advanced/Metastatic Solid Tumors or Relapsed/Refractory Hematologic Malignancies
Source: Cancer Res Commun. 2025 Nov 19;5(11):2025–38. doi: 10.1158/2767-9764.CRC-25-0243 (PMC12627933; doi:10.1158/2767-9764.CRC-25-0243)
Supplement: Supplementary Table 1 — Patient disposition and median duration of treatment (days) by subasumstat cohorts in phase I and II (safety population) [file crc-25-0243_supplementary_table_1_suppst1.pdf]

Supplementary Table 1. Patient disposition and median duration of treatment (days) by subasumstat cohorts in phase I and II (safety population).

Phase I – BIW									
**Subasumstat 3 mg**	**Subasumstat 6 mg**	**Subasumstat 10 mg**	**Subasumstat 15 mg**	**Subasumstat 25 mg**	**Subasumstat 40 mg**	**Subasumstat 60 mg**	**Subasumstat 75 mg**	**Subasumstat 90 mg**	**Subasumstat 120 mg**
***n* = 5**	***n* = 3**	***n* = 4**	***n* = 3**	***n* = 4**	***n* = 4**	***n* = 7**	***n* = 6**	***n* = 8**	***n* = 8**
11.0	22.0	36.0	32.0	44.5	156.5	31.0	32.0	18.0	32.0

Phase I – QW			
**Subasumstat 60 mg**	**Subasumstat 75 mg**	**Subasumstat 90 mg**	**Subasumstat 120 mg**
***n* = 6**	***n* = 6**	***n* = 7**	***n* = 6**
29.5	75.0	64.0	49.5

Phase I – days 1, 8 and 15
**Subasumstat 90 mg**
***n* = 7**
36.0

Phase II – BIW 90 mg					
**NSCLC**	**Cervical cancer**	**CRC**	**r/r DLBCL post-CART**	**r/r DLBCL**	**r/r FL**
***n* = 7**	***n* = 3**	***n* = 7**	***n* = 4**	***n* = 3**	***n* = 1**
121.0	116.0	32.0	33.5	12.0	338.0

BIW, twice weekly (days 1, 4, 8 and 11); CART, chimeric antigen receptor therapy; CRC, colorectal cancer, DLBCL, diffuse large B-cell lymphoma; FL, follicular lymphoma; NSCLC, non-small cell lung cancer; QW, weekly (days 1 and 8); r/r, relapsed refractory
