# Supplementary material for: A First-In-Human Study of the SUMOylation Inhibitor Subasumstat in Patients with Advanced/Metastatic Solid Tumors or Relapsed/Refractory Hematologic Malignancies
Source: Cancer Res Commun. 2025 Nov 19;5(11):2025–38. doi: 10.1158/2767-9764.CRC-25-0243 (PMC12627933; doi:10.1158/2767-9764.CRC-25-0243)
Supplement: Supplementary Table 2 — Representativeness of study participants. [file crc-25-0243_supplementary_table_2_suppst2.pdf]

Supplementary Table 2. Representativeness of study participants.

**Cancer type**	**Advanced metastatic solid and hematologic tumors**
**Considerations related to:**	
**Age**	The incidence of cancer diagnoses in the US increases with age for both males and females,[1,2] with cancer representing the leading cause of death among males aged between 60-79 years old and females aged 40-79 years old.[3]<br><br>The median age at which patients are diagnosed with cancer in the US is 67 years; the median age at death from cancer is 73 years.[1,2]
**Sex**	In the US, across all cancer types, both the incidence and the mortality rates due to cancer remain slightly higher in males (51–54% incidence, 53% mortality) versus females (46–49% incidence, 47% mortality).[3,4]<br><br>US males also have increased cumulative risk of developing cancer before the age of 75 (37.1%) than US females (31.7%).[4]
**Race/ethnicity**	People of Hispanic or Latino ethnicity represent 18.7% of the population in the US and constitute 7.4% of the population affected by cancer. Black or African American people represent 14.2% of the US population and constitute 10% of the population affected by cancer.[5,6]
**Geography**	In 2022, there were an estimated 2,380,189 new cases and 605,761 deaths attributed to cancer in the US; this respectively represents 12% and 6% of the global new cases and death.[4]<br><br>In 2022, the most frequent cancers in the US (>5%), across both sexes, were: breast (11.5%), prostate (9.7%), lung (9.5%) and colorectal (6.7%).[4]
**Overall representativeness of this study**	Overall, the median age in our phase I/II study is slightly lower than the median age at which patients in the US are diagnosed with cancer (62.5 years for Phase I and 61.0 years for Phase II vs 67 years).<br><br>The proportion of males and females in our study was respectively 44 and 56% in Phase I and 56% and 44% in Phase II. Based on the reported higher risk of cancer

	among males, the proportion of males in Phase I was lower than expected, but suitable in Phase II.<br><br>Overall, 9.5% of the study population identified as Black or African American, a suitable proportion based on the reported incidence of cancer in this population. Hispanic or Latino patients constituted 5% of the study population, a proportion slightly lower than expected based on the incidence of cancer in this patient population.

1. U.S. Cancer Statistics Working Group. U.S. Cancer Statistics Data Visualizations Tool. U.S. Department of Health and Human Services, Centers for Disease Control and Prevention and National Cancer Institute; https://www.cdc.gov/cancer/dataviz , released in June 2024. Accessed [29 Jan 2025].
2. SEER Explorer: An interactive website for SEER cancer statistics. Surveillance Research Program, National Cancer Institute; 2024 Apr 17. [updated: 2024 Nov 5; cited 2025 Jan 29]. Available from: https://seer.cancer.gov/statistics-network/explorer/. Data source(s): SEER Incidence Data, November 2023 Submission (1975-2021), SEER 22 registries. Accessed [29 Jan 2025]
3. Siegel, R.L., Giaquinto, A.N. & Jemal, A. Cancer statistics, 2024. CA Cancer J Clin 74, 12-49 (2024).
4. Ferlay J, Ervik M, Lam F, Laversanne M, Colombet M, Mery L, Piñeros M, Znaor A, Soerjomataram I, Bray F (2024). Global Cancer Observatory: Cancer Today. Lyon, France: International Agency for Research on Cancer. Available from: https://gco.iarc.who.int/today, accessed [22 Jan 2025].
5. Jones N, Marks R, Ramirez R, Rios-Vargas M (2021). 2020 Census Illuminates Racial and Ethnic Composition of the Country. https://www.census.gov/library/stories/2021/08/improved-race-ethnicity-measures-reveal-united-states-population-much-more-multiracial.html , accessed [29 Jan 2025]
6. National Cancer Institute. SEER cancer statistics review, 1975-2017 - Table 18.21, 2021. https://seer.cancer.gov/archive/csr/1975_2017/results_merged/sect_18_myeloma.pdf. Accessed [29 Jan 2025]
