# Supplementary material for: A First-In-Human Study of the SUMOylation Inhibitor Subasumstat in Patients with Advanced/Metastatic Solid Tumors or Relapsed/Refractory Hematologic Malignancies
Source: Cancer Res Commun. 2025 Nov 19;5(11):2025–38. doi: 10.1158/2767-9764.CRC-25-0243 (PMC12627933; doi:10.1158/2767-9764.CRC-25-0243)
Supplement: Supplementary Table 3 — Dose limiting toxicities with single agent subasumstat. [file crc-25-0243_supplementary_table_3_suppst3.pdf]

Supplementary Table 3. Dose limiting toxicities with single agent subasumstat.

Subasumstat dose level	*N*	*n* with DLTs during cycle 1	Description of TEAEs	Action taken
60 mg BIW	6	1	Transient grade 3 ALT/AST elevation	Subasumstat reduced to 40 mg BIW
90 mg BIW	6	1	Grade 3 pneumonitis	Study treatment discontinued
120 mg BIW	6	2	Grade 3 stomatitis (*n* = 1)	Subasumstat reduced to 90 mg BIW
			Grade 3 cognitive disorder (*n* = 1)	Study treatment discontinued

ALT, alanine aminotransferase; AST, aspartate aminotransferase; BIW, twice weekly; DLT, dose-limiting toxicity; TEAE, treatment-emergent adverse event.
