# Supplementary material for: A First-In-Human Study of the SUMOylation Inhibitor Subasumstat in Patients with Advanced/Metastatic Solid Tumors or Relapsed/Refractory Hematologic Malignancies
Source: Cancer Res Commun. 2025 Nov 19;5(11):2025–38. doi: 10.1158/2767-9764.CRC-25-0243 (PMC12627933; doi:10.1158/2767-9764.CRC-25-0243)
Supplement: Supplementary Table 4 — Subasumstat-related TEAEs occurring in ≥5% of all patients. [file crc-25-0243_supplementary_table_4_suppst4.pdf]

Supplementary Table 4. Subasumstat-related TEAEs occurring in ≥5% of all patients.

TEAEs, preferred term *n* (%)	Phase I (*n* = 84)	Phase II (*n* = 25)	Total (*n* = 109)
Fatigue	27 (32.1)	7 (28.0)	34 (31.2)
Nausea	29 (34.5)	4 (16.0)	33 (30.3)
Pyrexia	23 (27.4)	9 (36.0)	32 (29.4)
Headache	21 (25.0)	5 (20.0)	26 (23.9)
Chills	17 (20.2)	6 (24.0)	23 (21.1)
Diarrhea	15 (17.9)	6 (24.0)	21 (19.3)
Vomiting	13 (15.5)	1 (4.0)	14 (12.8)
Decreased appetite	10 (11.9)	3 (12.0)	13 (11.9)
CRS	9 (10.7)	3 (12.0)	12 (11.0)
Anemia	4 (4.8)	4 (16.0)	8 (7.3)
Myalgia	6 (7.1)	2 (8.0)	8 (7.3)
Hypokalemia	7 (8.3)	0	7 (6.4)
IRR	5 (6.0)	1 (4.0)	6 (5.5)
Dyspnea	4 (4.8)	2 (8.0)	6 (5.5)

CRS, cytokine release syndrome; IRR, infusion-related reaction; TEAE, treatment-emergent adverse event.
