# Supplementary material for: A First-In-Human Study of the SUMOylation Inhibitor Subasumstat in Patients with Advanced/Metastatic Solid Tumors or Relapsed/Refractory Hematologic Malignancies
Source: Cancer Res Commun. 2025 Nov 19;5(11):2025–38. doi: 10.1158/2767-9764.CRC-25-0243 (PMC12627933; doi:10.1158/2767-9764.CRC-25-0243)
Supplement: Supplementary Table 5 — Grade ≥3 subasumstat-related TEAEs occurring in ≥1% of all patients. [file crc-25-0243_supplementary_table_5_suppst5.pdf]

Supplementary Table 5. Grade ≥3 subasumstat-related TEAEs occurring in ≥1% of all patients.

**Grade ≥3 TEAEs, preferred term** *n* **(%)**	**Phase I (*n* = 84)**	**Phase II (*n* = 25)**	**Total (*n* = 109)**
Patients with at least one grade ≥3 TEAE	18 (21.4)	11 (44.0)	29 (26.6)
Anemia	1 (1.2)	3 (12.0)	4 (3.7)
Neutropenia	0	4 (16.0)	4 (3.7)
Lymphocyte count decreased	4 (4.8)	0	4 (3.7)
Platelet count increased	1 (1.2)	2 (8.0)	3 (2.8)
Hypokalemia	2 (2.4)	0	2 (1.8)
Thrombocytopenia	1 (1.2)	1 (4.0)	2 (1.8)
Aspartate aminotransferase increased	2 (2.4)	0	2 (1.8)
Pneumonitis	2 (2.4)	0	2 (1.8)
Muscular weakness	0	2 (8.0)	2 (1.8)

TEAE, treatment-emergent adverse event.
