# Supplementary material for: A First-In-Human Study of the SUMOylation Inhibitor Subasumstat in Patients with Advanced/Metastatic Solid Tumors or Relapsed/Refractory Hematologic Malignancies
Source: Cancer Res Commun. 2025 Nov 19;5(11):2025–38. doi: 10.1158/2767-9764.CRC-25-0243 (PMC12627933; doi:10.1158/2767-9764.CRC-25-0243)
Supplement: Supplementary Figure 1 — Subasumstat mechanism of action. [file crc-25-0243_supplementary_figure_1_suppsf1.pdf]

Supplementary Figure 1. Subasumstat mechanism of action

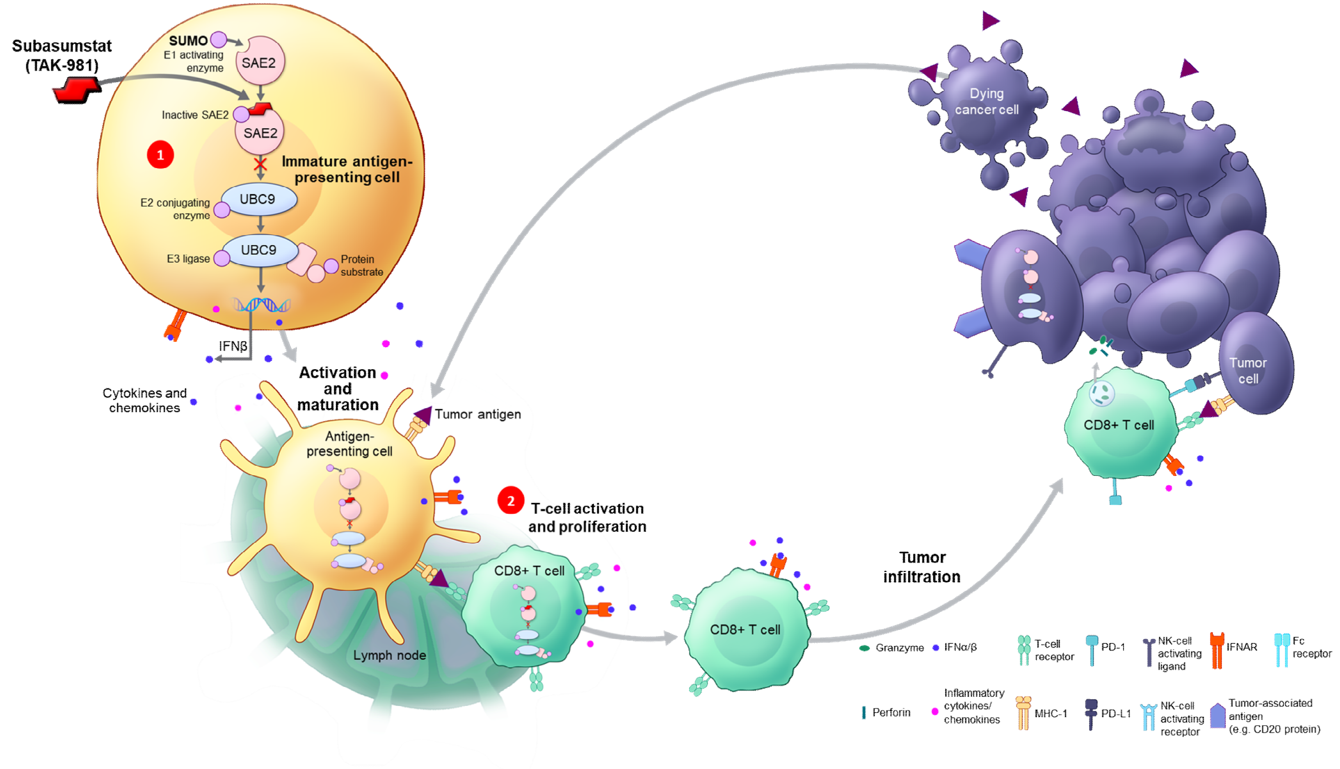

1) Subasumstat inhibits SUMO-activating enzyme (SAE), restoring endogenous type 1 interferon (α/β) signaling and thereby stimulating APCs, NK cells, T cells, and macrophages. 2) Subasumstat enhances antigen presentation and T-cell activation and tumor infiltration.

IFNAR, interferon receptor; MHC, major histocompatibility complex; PD-1, programmed cell death protein 1; PD-L1, programmed death-ligand 1; SUMO; small ubiquitin-like modifier; UBC, ubiquitin-conjugating enzyme.
