# Supplementary material for: A First-In-Human Study of the SUMOylation Inhibitor Subasumstat in Patients with Advanced/Metastatic Solid Tumors or Relapsed/Refractory Hematologic Malignancies
Source: Cancer Res Commun. 2025 Nov 19;5(11):2025–38. doi: 10.1158/2767-9764.CRC-25-0243 (PMC12627933; doi:10.1158/2767-9764.CRC-25-0243)
Supplement: Supplementary Figure 2 — Mean plasma concentration over time profiles of subasumstat – phase II. [file crc-25-0243_supplementary_figure_2_suppsf2.pdf]

Supplementary Figure 2. Mean plasma concentration over time profiles of subasumstat – phase II.

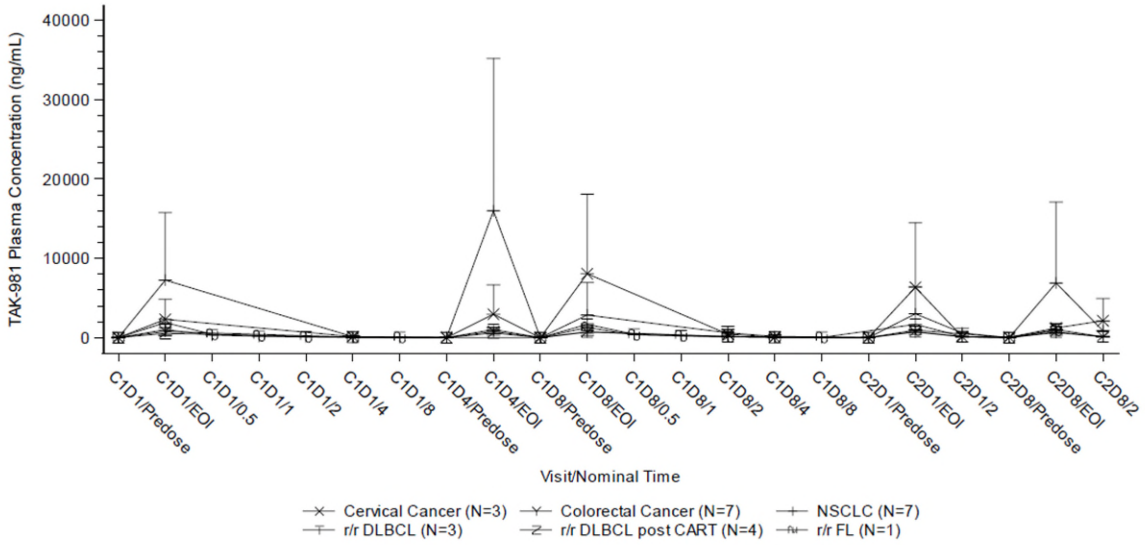

Linear scale.

C1D1, cycle 1 day; CAR-T, chimeric antigen receptor T cell; DLBCL, diffuse large B-cell lymphoma; EOI, end of infusion; FL, follicular lymphoma; IV, intravenous; NSCLC, non-small cell lung cancer; r/r, relapsed/refractory.
