# Supplementary material for: A First-In-Human Study of the SUMOylation Inhibitor Subasumstat in Patients with Advanced/Metastatic Solid Tumors or Relapsed/Refractory Hematologic Malignancies
Source: Cancer Res Commun. 2025 Nov 19;5(11):2025–38. doi: 10.1158/2767-9764.CRC-25-0243 (PMC12627933; doi:10.1158/2767-9764.CRC-25-0243)
Supplement: Supplementary Figure 3 — Subasumstat-SUMO adduct formation in peripheral blood lymphocytes. [file crc-25-0243_supplementary_figure_3_suppsf3.pdf]

Supplementary Figure 3. Subasumstat-SUMO adduct formation in peripheral blood lymphocytes (A) and skin biopsies (B) following subasumstat BIW and QW administration (all doses) – phase I (pharmacodynamic population).

A

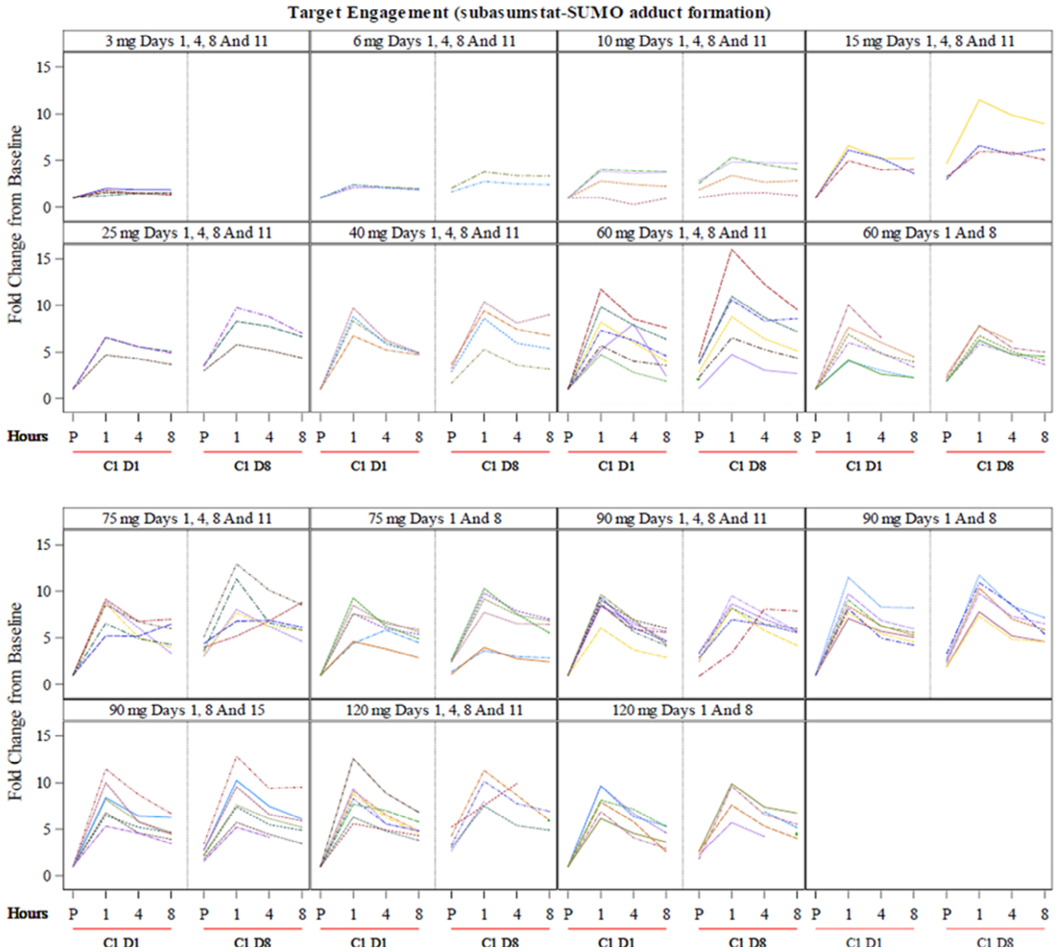

B

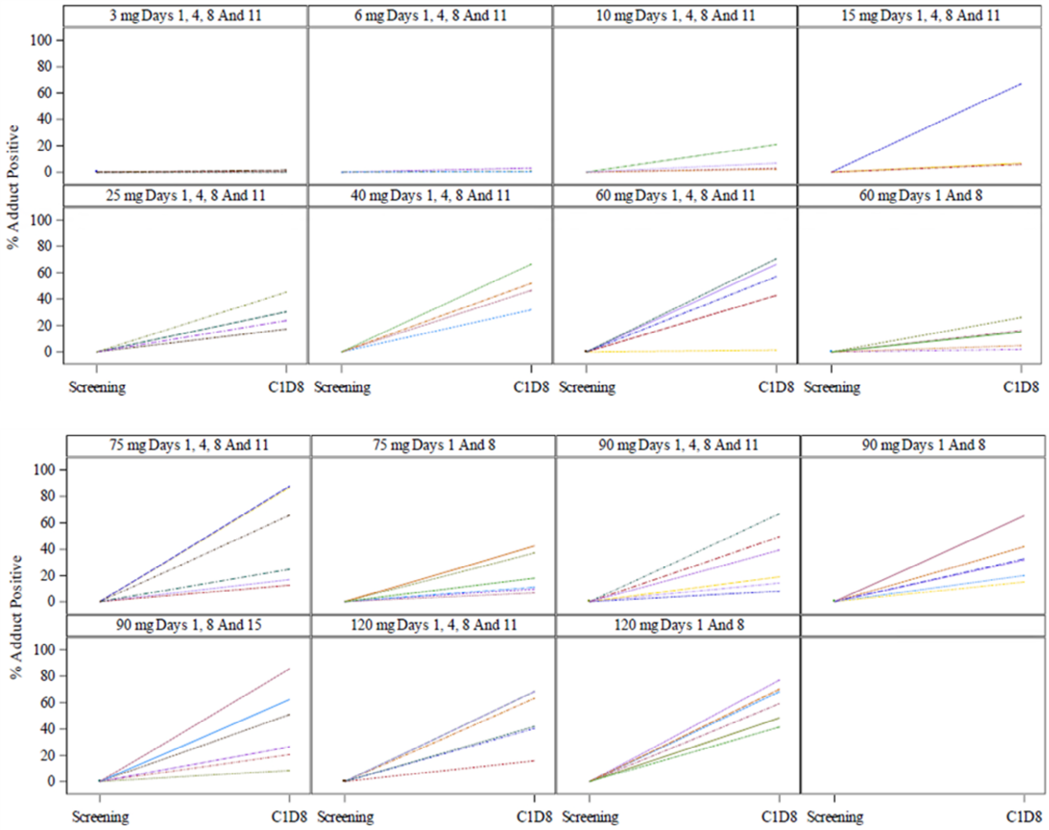

BIW, twice weekly (days 1, 4, 8, and 11); QW, weekly (days 1 and 8); SUMO, small ubiquitin-like modifier.
