# Supplementary material for: A First-In-Human Study of the SUMOylation Inhibitor Subasumstat in Patients with Advanced/Metastatic Solid Tumors or Relapsed/Refractory Hematologic Malignancies
Source: Cancer Res Commun. 2025 Nov 19;5(11):2025–38. doi: 10.1158/2767-9764.CRC-25-0243 (PMC12627933; doi:10.1158/2767-9764.CRC-25-0243)
Supplement: Supplementary Figure 7 — Activation of IFN-I pathway following subasumstat 90 mg BIW administration – phase II. [file crc-25-0243_supplementary_figure_7_suppsf7.pdf]

Supplementary Figure 7. Activation of IFN-I pathway following subasumstat 90 mg BIW administration – phase II.

A

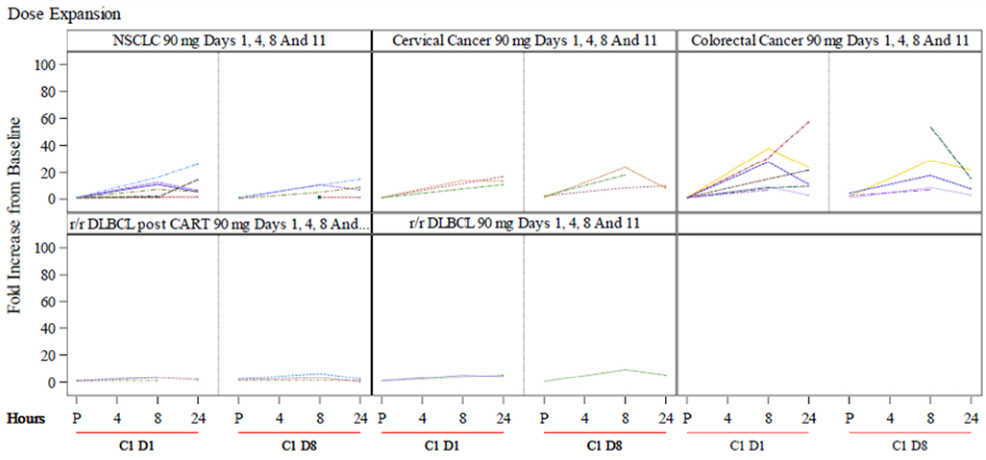

B

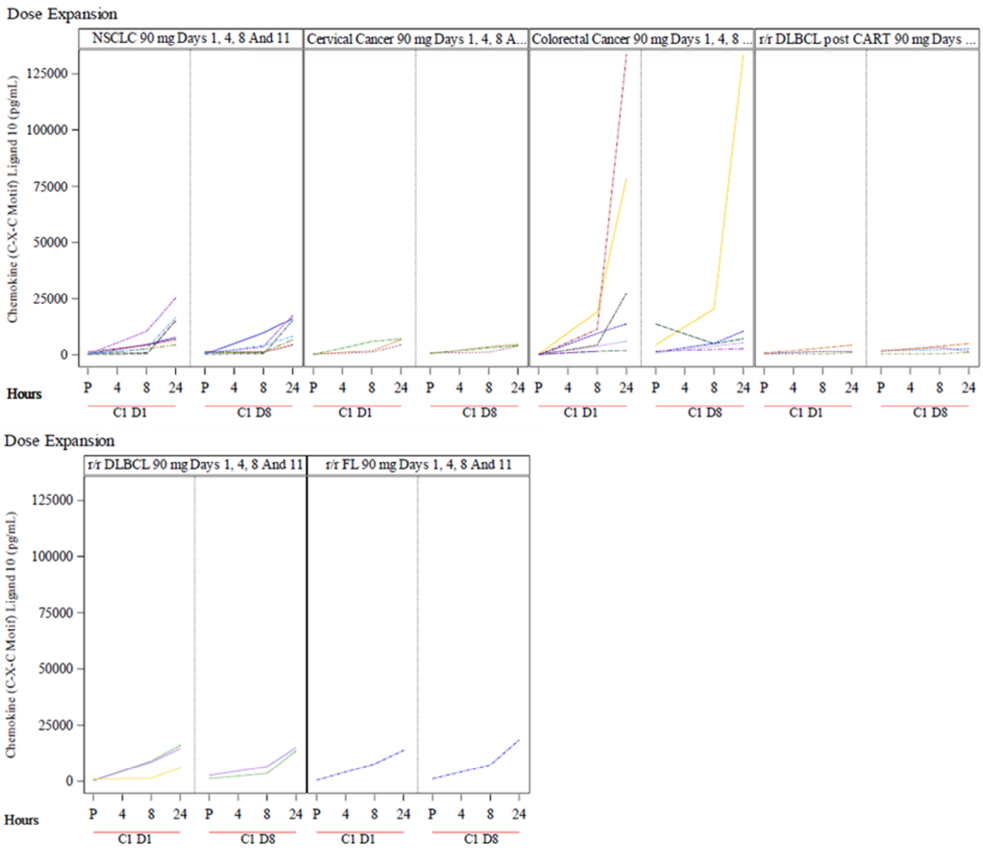

C

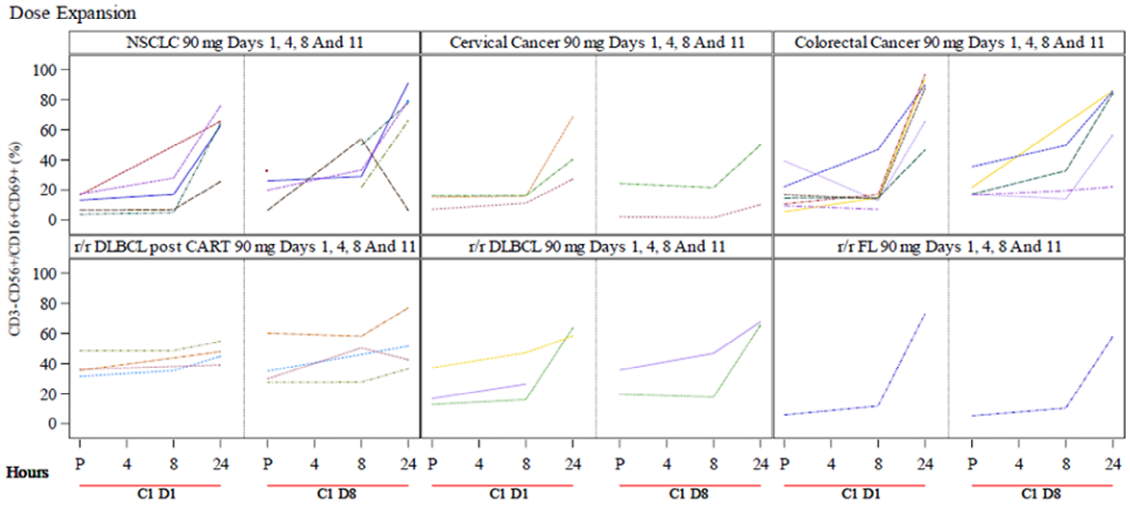

D

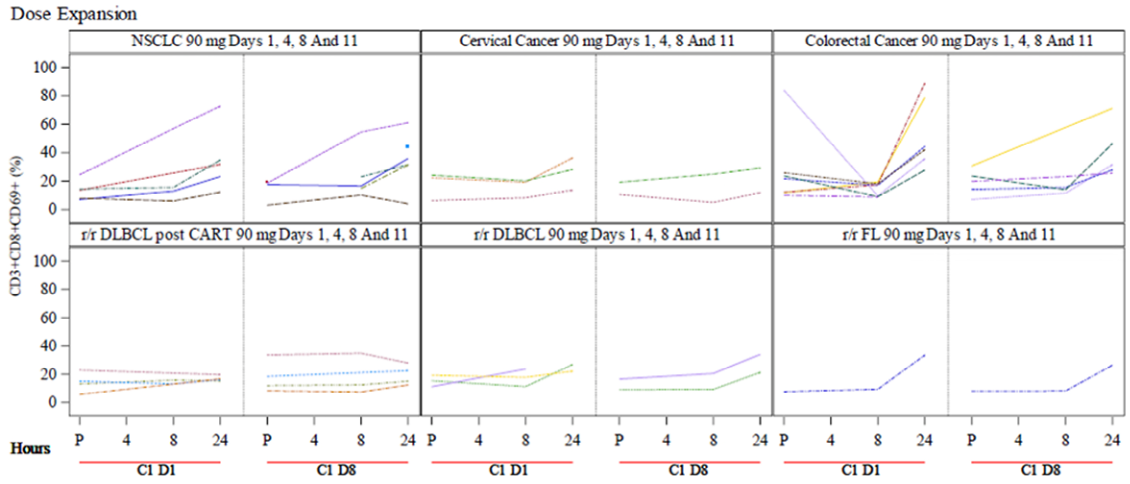

A) IFN-I gene signature in peripheral blood at selected doses
B) CXCL-10 levels in plasma at selected doses
C) Percentage of CD69-positive cells in NK cells (CD3-CD56+/CD16+CD69+ %) at selected doses
D) Percentage of CD69-positive cells in CD8+ T cells (CD3-CD56+/CD16+CD69+ %) at selected doses

BIW, twice weekly (days 1, 4, 8, and 11); IFN, interferon; NK, natural killer.
