# Supplementary material for: A First-In-Human Study of the SUMOylation Inhibitor Subasumstat in Patients with Advanced/Metastatic Solid Tumors or Relapsed/Refractory Hematologic Malignancies
Source: Cancer Res Commun. 2025 Nov 19;5(11):2025–38. doi: 10.1158/2767-9764.CRC-25-0243 (PMC12627933; doi:10.1158/2767-9764.CRC-25-0243)
Supplement: Supplementary Figure 8 — Multiplex immunofluorescence analysis of tumor biopsies at screening and following subasumstat administration. [file crc-25-0243_supplementary_figure_8_suppsf8.pdf]

Supplementary Figure 8. Multiplex immunofluorescence analysis of tumor biopsies at screening and following subasumstat administration.

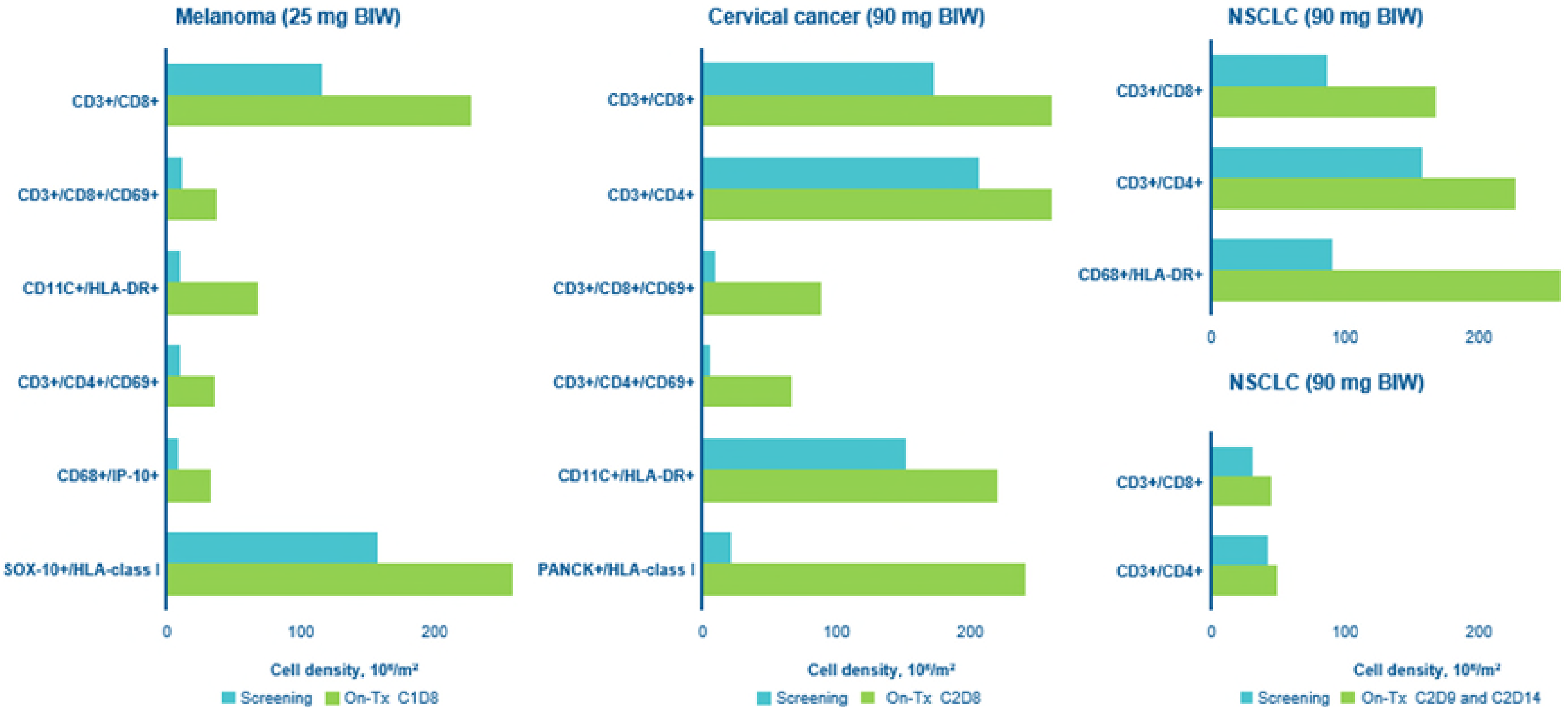

Graphs represent data from one patient. There was one patient for melanoma and cervical cancer each, and two patients for NSCLC. BIW, twice weekly (days 1, 4, 8, and 11); NSCLC, non-small cell lung cancer.
