# Supplementary material for: A First-In-Human Study of the SUMOylation Inhibitor Subasumstat in Patients with Advanced/Metastatic Solid Tumors or Relapsed/Refractory Hematologic Malignancies
Source: Cancer Res Commun. 2025 Nov 19;5(11):2025–38. doi: 10.1158/2767-9764.CRC-25-0243 (PMC12627933; doi:10.1158/2767-9764.CRC-25-0243)
Supplement: Supplementary Figure 9 — Effect of subasumstat on TME and PD-L1 expression in tumor biopsies. [file crc-25-0243_supplementary_figure_9_suppsf9.pdf]

Supplementary Figure 9. Effect of subasumstat on TME and PD-L1 expression in tumor biopsies.

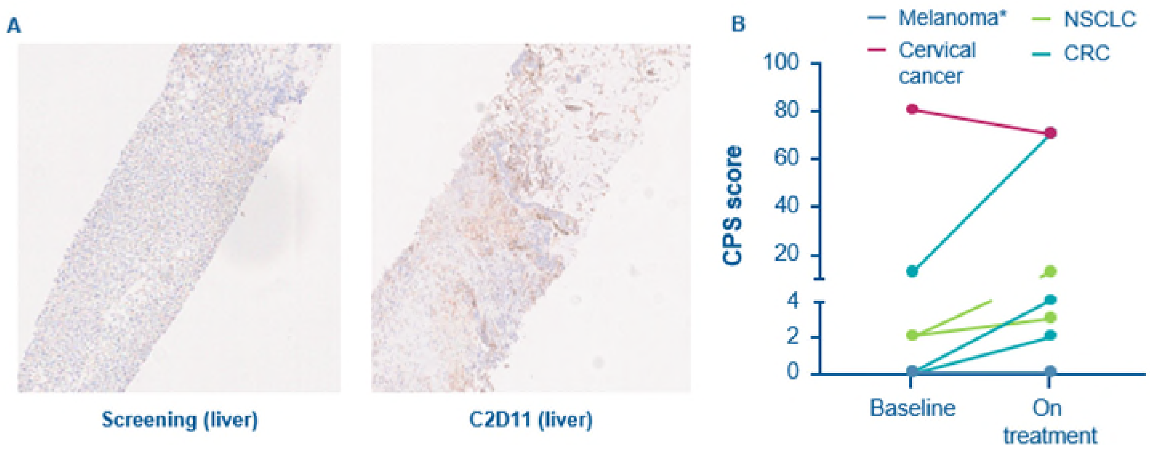

A) Representative IHC of liver metastasis sample from a patient in the CRC cohort at screening and at C2D11 following treatment with subasumstat 90 mg BIW, using anti-PD-L1 22C3 assay
B) Quantification of PD-L1 CPS.

*All data from phase II, following subasumstat 90 mg BIW dose on C2D8 except melanoma, which was from phase I, following subasumstat 25 mg BIW dose on C1D8. Tumor samples were collected on C2D8 for cervical cancer, C2D9 or C2D14 for NSCLC, and C2D10 or C2D15 for CRC.

BIW, twice weekly (days 1, 4, 8, and 11); C2D11, cycle 2 day 11; CPS, combined positive score; CRC, colorectal cancer; IHC, immunohistochemistry; NSCLC, non-small cell lung cancer; PD-L1, programmed death-ligand 1.
