# Supplementary material for: A First-In-Human Study of the SUMOylation Inhibitor Subasumstat in Patients with Advanced/Metastatic Solid Tumors or Relapsed/Refractory Hematologic Malignancies
Source: Cancer Res Commun. 2025 Nov 19;5(11):2025–38. doi: 10.1158/2767-9764.CRC-25-0243 (PMC12627933; doi:10.1158/2767-9764.CRC-25-0243)
Supplement: Supplementary Figure 10 — Swimmer plot of patient responses. [file crc-25-0243_supplementary_figure_10_suppsf10.pdf]

Supplementary Figure 10. Swimmer plot of patient responses (Safety population) – phases I (A) and II (B).

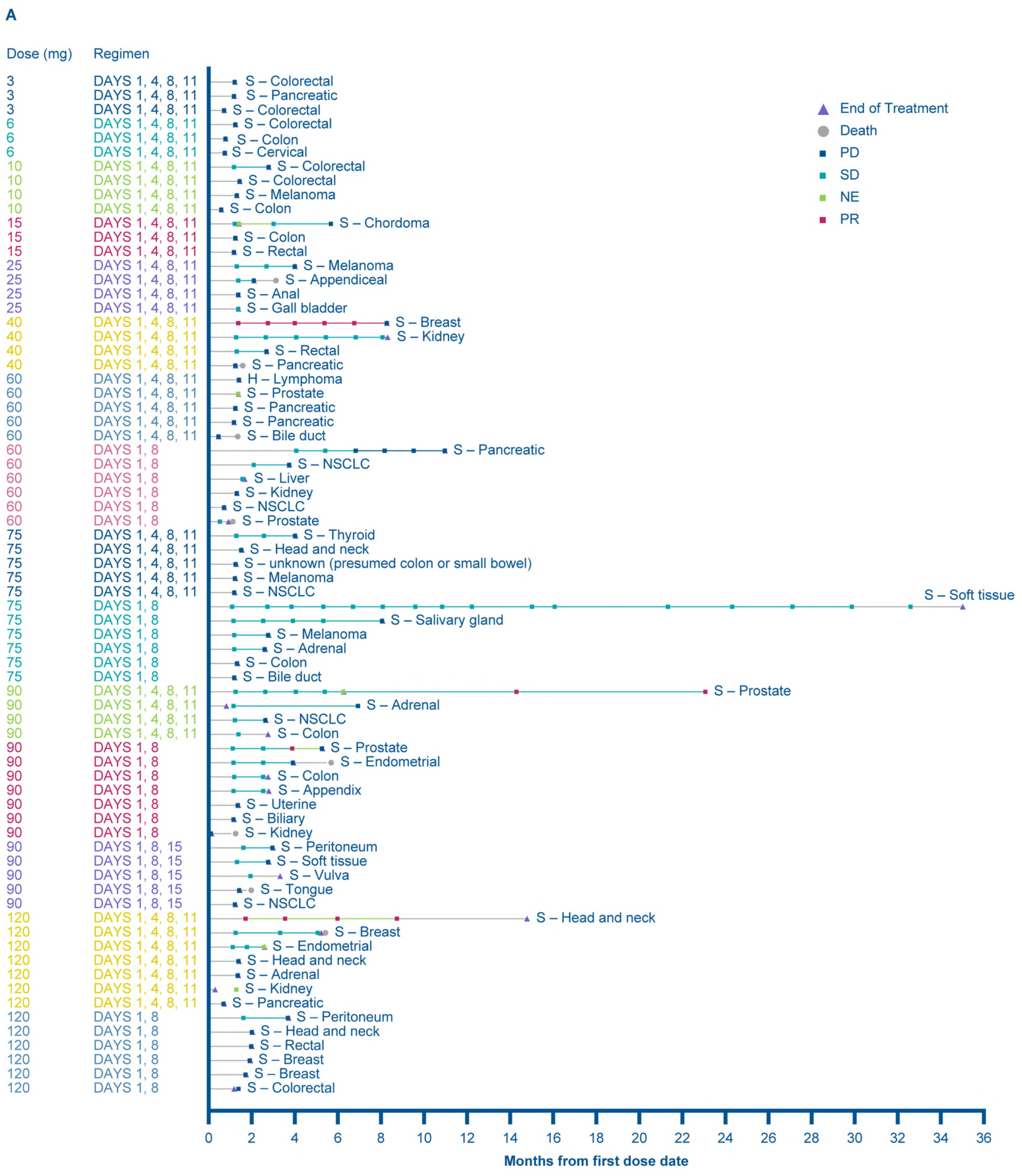

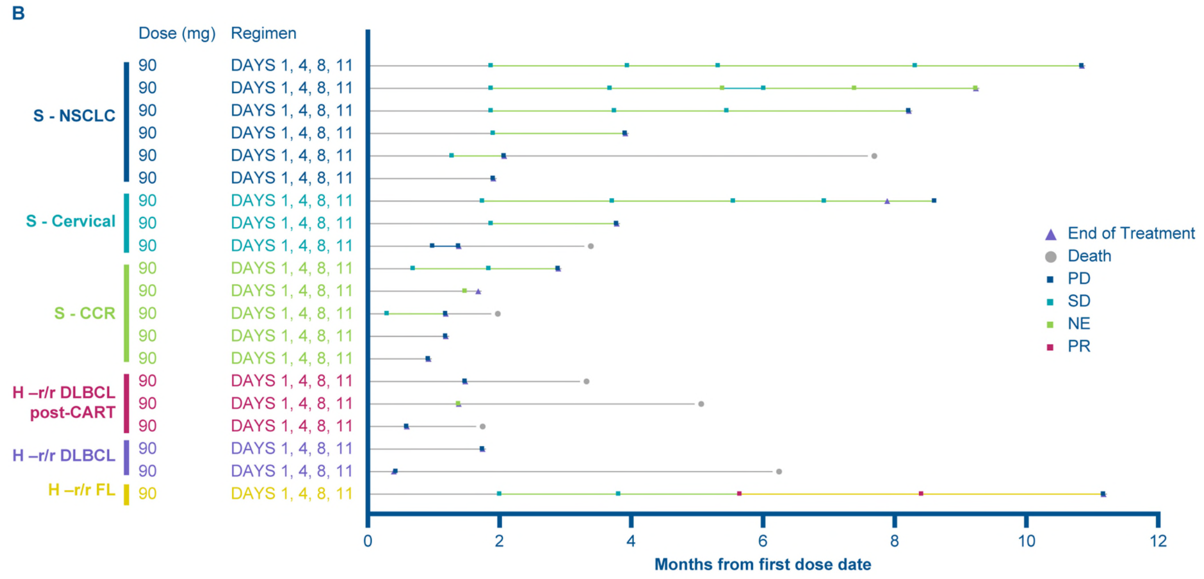

H, hematologic; CART, chimeric antigen receptor T-cell therapy; DLBCL, diffuse large B-cell lymphoma; FL, follicular lymphoma; NE, not estimated; NSCLC, non-small cell lung cancer; PD, disease progression; PR, partial response; r/r, relapsed/refractory; S, solid; SD, stable disease
